# Purifying Selection on Splice-Related Motifs, Not Expression Level nor RNA Folding, Explains Nearly All Constraint on Human lincRNAs

**DOI:** 10.1093/molbev/msu249

**Published:** 2014-08-25

**Authors:** Andreas Schüler, Avazeh T. Ghanbarian, Laurence D. Hurst

**Affiliations:** ^1^Institute for Evolution and Biodiversity, University of Münster, Münster, Germany; ^2^Department of Biology and Biochemistry, University of Bath, Bath, United Kingdom

**Keywords:** ncRNA, rate of evolution, splicing

## Abstract

There are two strong and equally important predictors of rates of human protein evolution: The amount the gene is expressed and the proportion of exonic sequence devoted to control splicing, mediated largely by selection on exonic splice enhancer (ESE) motifs. Is the same true for noncoding RNAs, known to be under very weak purifying selection? Prior evidence suggests that selection at splice sites in long intergenic noncoding RNAs (lincRNAs) is important. We now report multiple lines of evidence indicating that the great majority of purifying selection operating on lincRNAs in humans is splice related. Splice-related parameters explain much of the between-gene variation in evolutionary rate in humans. Expression rate is not a relevant predictor, although expression breadth is weakly so. In contrast to protein-coding RNAs, we observe no relationship between evolutionary rate and lincRNA stability. As in protein-coding genes, ESEs are especially abundant near splice junctions and evolve slower than non-ESE sequence equidistant from boundaries. Nearly all constraint in lincRNAs is at exon ends (N.B. the same is not witnessed in *Drosophila*). Although we cannot definitely answer the question as to why splice-related selection is so important, we find no evidence that splicing might enable the nonsense-mediated decay pathway to capture transcripts incorrectly processed by ribosomes. We find evidence consistent with the notion that splicing modifies the underlying chromatin through recruitment of splice-coupled chromatin modifiers, such as CHD1, which in turn might modulate neighbor gene activity. We conclude that most selection on human lincRNAs is splice mediated and suggest that the possibility of splice–chromatin coupling is worthy of further scrutiny.

## Introduction

Understanding how genes evolve and where purifying selection is acting to maintain the status quo can, in principle, be highly informative of the function of a gene and the reasons that mutations might be deleterious and potentially causative of disease. In the simplest instance, for example, selection to preserve functional protein motifs is commonly taken to imply a function for that motif and possible pathogenic consequences for mutations that disrupt the motif. On a broad scale, we can approach these issues by asking where in genes we see purifying selection and what determines the variation between genes in their rate of evolution. Although the determinants of the rate of protein evolution are much studied (Pal et al. 2006; Zeldovich and Shakhnovich 2008), much less well understood are the determinants of the evolutionary rate of noncoding RNAs (ncRNAs). The exons of human ncRNAs are typically poorly conserved compared with protein-coding genes (Marques and Ponting 2009) and on average evolve a little slower than their flanking introns (Hurst and Smith 1999; Pang et al. 2006), suggesting weak purifying selection. The causes of this are unclear (Pang et al. 2006). The relatively rapid evolution need not imply an absence of function, as even highly functional ncRNAs, such as *Xist*, contain only a few conserved stretches (Pang et al. 2006). The determinants of between-gene variation in the rate of evolution of ncRNAs are only beginning to be explored (Managadze et al. 2011). Here then we ask about where in ncRNAs purifying selection operates and what predicts rates of evolution of ncRNAs.

Understanding the evolution of ncRNA can, conversely, potentially shed important light on the mode of selection on protein-coding genes. For example, it has recently been suggested that selection on RNA stability is an important determinant of rate of protein evolution (Park et al. 2013). It is, however, unknown whether this selection is particular to RNAs that are translated or to all RNA species. In principle, one can imagine models for either possibility. For example, RNA stability selection may be important in altering translational dynamics if RNA structure modulates ribosomal speed. Conversely, the selection may simply be to enable RNA to persist in a stable configuration, in which case ncRNA might be under similar selection. For proteins there is at least one universal predictor of between-gene variation in rate of evolution, namely the more a gene is expressed the lower its evolutionary rate (Pal et al. 2001; Drummond et al. 2006). One hypothesis to explain this concerns selection on protein-folding accuracy (Drummond and Wilke 2008; Yang et al. 2010). If the correlation between protein rate of expression and rate of evolution is mediated exclusively by selection on protein folding, then we expect no such correlation in ncRNAs. One prior claim (Managadze et al. 2011) identified slower evolution in more highly expressed ncRNA and found a coupling between long intergenic ncRNA (lincRNA) stability and evolutionary rate. They concluded there to be a universal (all transcript types) correlation between expression level and evolutionary rate. This they took to suggest a possible universal selection on folding, be it RNA or protein level. Given the importance of such a result, we now return to this issue.

In mammals, splice-related constraints are of an approximately equal magnitude to the expression-related parameters as a predictor of rate of protein evolution (Parmley et al. 2007). Although splice sites are necessary for exon–intron junction recognition, they carry only some of the information required for accurate splicing of protein-coding genes (Lim and Burge 2001). Exonic splice enhancers (ESEs) are also necessary to maintain proper splicing. ESE motifs are purine-rich hexamers that bind serine arginine-rich (SR) proteins to aid exonic splice site recognition (Blencowe 2000; Cartegni et al. 2002). They mostly operate close to (within 70 bp) exon–intron junctions (Fairbrother, Holste, et al. 2004) in a quantitative fashion, such that the higher the density of ESEs the higher the splice rate (Graveley 2000; Fairbrother et al. 2002; Fairbrother, Holste, et al. 2004; Fairbrother, Yeo, et al. 2004; Ke et al. 2011). On average 30–40% of bases at the flanks of protein-coding exons feature in at least one experimentally confirmed motif, this proportion being higher for exons flanked by larger introns (Dewey et al. 2006) where exon definition is especially difficult.

Owing to their abundance, importance, and skewed nucleotide content, ESEs leave strong and easily identified footprints in the molecular evolution of mammalian protein-coding genes (Cáceres and Hurst 2014). ESE motifs evolve at considerably lower rates than non-ESE sites, at both the synonymous (Carlini and Genut 2006; Parmley et al. 2006) and nonsynonymous levels (Parmley et al. 2007). The abundance of ESEs near exon junctions skews amino acid content and codon usage patterns (Parmley and Hurst 2007; Parmley et al. 2007), with the majority of amino acids and codons showing avoidance or preference near boundaries, these trends being well predicted by ESE nucleotide content. As “boundary” regions are large with respect to the average size of an exon, the biology of ESEs is one of the major influences on human protein-coding genes.

ncRNAs frequently contain conserved promoter regions and splice sites and also show a reduced rate of insertions and deletions (Ponjavic et al. 2007), indicative of selection for splicing and transcription. Indeed, conserved splice sites have been employed to identify noncoding transcripts (Rose et al. 2011) and splice sites in ncRNA often show considerable degrees of conservation (Nitsche et al. 2014; Washietl et al. 2014). It is, however, unknown whether ESEs are involved in splice regulation and, assuming that they are, whether they contribute to purifying selection operating on sequence. Here then we employ a robust and appropriate high-quality data set of human ncRNAs (Cabili et al. 2011), wherein we can both have a good measure of confidence that the ncRNAs are not protein coding, that the ncRNA are real (by them being identified more than once), and that, being intergenic, there are minimal issues with overlapping transcripts. Of these data, we ask 1) whether ncRNAs show evidence of splice-related constraint with reduced rates of evolution at exonic ends, especially in residues associated with exonic splice enhancer motifs; 2) if so, what proportion of the reduced rate of evolution of ncRNA exons, when compared with flanking introns, can be explained as owing to splice-related selection; and 3) how important is splice-related selection in explaining between-gene variation in rate of evolution of ncRNAs compared with other possible predictors. We report that the great majority of selection on ncRNAs is splice related, purifying selection being dominantly on exon ends with ESE motifs especially slow evolving. We consider a series of models to explain this unexpected result.

## Results

Do lincRNAs employ ESEs? If they do, can we find evidence for splice-related constraints within the exons on lincRNAs? If we can, how important are splice-related constraints, both in explaining any purifying selection operating on lincRNAs exons when compared with their introns and in explaining between-gene variation in rates of exonic evolution? To address the former issues, we start by considering whether exons of lincRNAs use ESEs in the same manner as protein-coding genes and in turn whether they impose comparable degrees of constraint.

### ESE Usage and within-Gene Variation in Rate of Evolution

#### ESE Usage at lincRNA Exonic Flanks Resembles That in Protein-Coding Exons

**Fig. 1.**
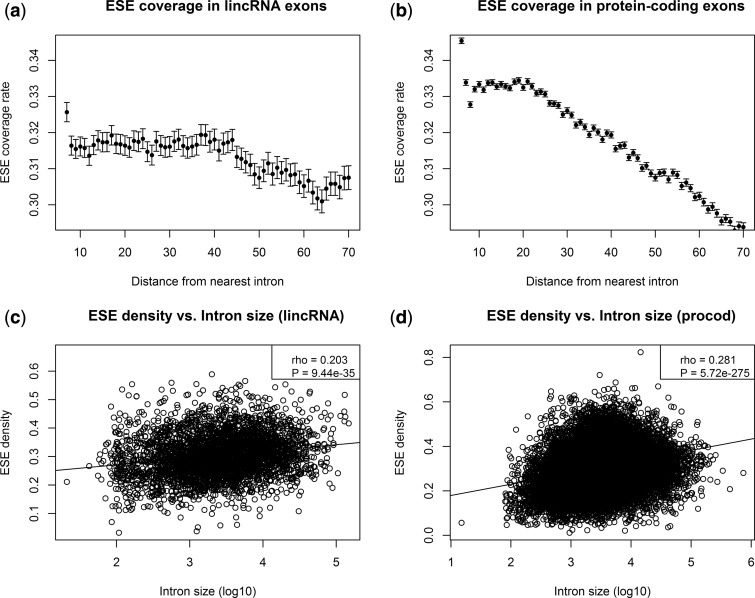
Relative frequencies of bases ± SEM predicted to be part of an ESE motif as a function of the distance to the nearest intron, starting at a distance of 6 [(*a*) and (*b*)]. The decadic logarithm of the average intron length for lincRNA and protein-coding genes versus the density of ESE motifs on the exon sequences of this gene is shown in (*c*) and (*d*). For (*c*) and (*d*), “density” has been measured as the number of nucleotides that belong to a putative ESE motif divided by the summed length of exons for the respective gene. This figure includes only the conservative lincRNAs. For the complete set, see supplementary figure S1, Supplementary Material online. Error bars for (*a*) and (*b*) = ± SEM.

ESEs are most efficient close to the splice junction (Fairbrother et al. 2002; Fairbrother, Holste, et al. 2004; Fairbrother, Yeo, et al. 2004) and if ESEs are involved in splicing regulation for lincRNAs, putative ESE motifs should be enriched close to splice junctions. To test this hypothesis, we annotated putative ESE motifs in the lincRNA and protein-coding alignments by using the set of experimentally confirmed human ESE-hexamers employed in a previous study (Parmley et al. 2006) as defined by Fairbrother, Yeo, et al. (2004). We temporarily removed gaps from the alignments to scan for matches to the set of known ESE-hexamers. Matching hexamers were masked and gaps were reinserted after the scan. As expected, the density of putative ESE motifs is highest in direct proximity to the splice sites and decreases with distance from the splice site. This trend is observed in both lincRNA and protein-coding exons (fig. 1*a* and *b*).

It has been shown that large introns are correlated with a high density of ESEs in the flanking exons (Dewey et al. 2006; Cáceres and Hurst 2014). We can reproduce this observation for both the protein-coding genes and the lincRNAs in our data set (fig. 1*c* and *d*). The trend is weaker in lincRNAs compared with protein-coding genes (rho = 0.2 and 0.28, respectively) but both correlations are highly significant (from Spearman: *P* < 10^−^^16^). Using all lincRNAs instead of only the conservative subset does not qualitatively change these results (supplementary fig. S1, Supplementary Material online).

#### Exonic Splice Enhancers Evolve Considerably Slower Than Nonenhancers in lincRNA Evolution

In protein-coding genes, exon residues that specify ESEs evolve slower than non-ESE sequence (Parmley et al. 2006, 2007). Our data replicate this result (median *K* [ESE] = 0.021, median *K* [non-ESE] = 0.028, Wilcoxon test: *P* < 10^−^^16^). More importantly, we find that substitution rates in ESEs are significantly lower than the ones in non-ESE sites in lincRNAs (median *K* [ESE] = 0.055, median *K* [non-ESE] = 0.066, Wilcoxon test: *P* < 10^−^^16^).

**Fig. 2.**
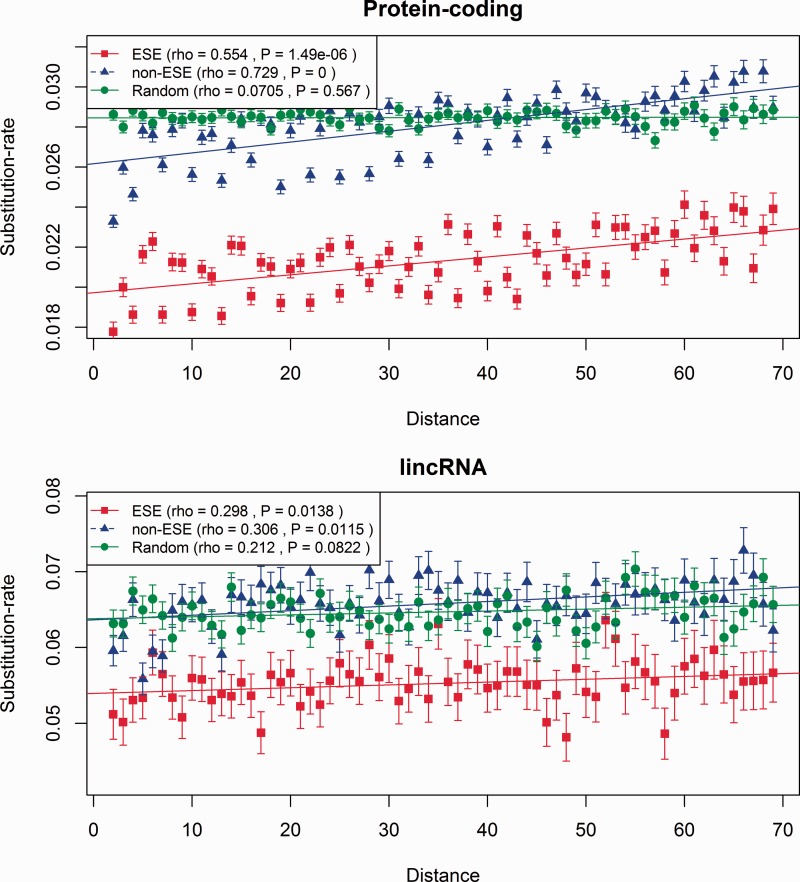
ESE motifs evolve slower than non-ESE sites. The substitution rates (number of substitutions divided by number of sites) in ESEs and non-ESEs are shown as a function of the distance in base pairs from the nearest splice-junction, for lincRNA (bottom) and protein-coding (top) genes. This figure includes only the conservative lincRNAs. For the complete set, see supplementary figure S2, Supplementary Material online. Bars indicate ± SEM.

Given that ESEs function close to exon boundaries, it might in turn be helpful to control for distance from an exon boundary. We thus compared the evolutionary rates between ESE and non-ESE sites as a function of the distance from the nearest splice junction. Conceptually, every exon was split in half and for each alignment site we assigned the base pair-distance to the 5′-splice junction for the first exon-half or to 3′-junction for the second exon-half. We calculated the substitution rates for sites up to 70 bp away from the nearest splice junction and distinguished between ESE and non-ESE for both protein coding RNAs (fig. 2*a*) and lincRNA (fig .2*b*). We again observe a trend of ESE sites evolving slower than non-ESE sites, and also a positional effect with the average substitution rates increasing with the distance from the nearest splice junction. This positional effect is observed both in lincRNA and in protein-coding genes (fig. 2). Using all lincRNAs instead of the conservative subset again does not qualitatively affect the results (supplementary fig. S2, Supplementary Material online).

ESEs are purine-rich and, as ESE density is also decreasing with distance from the nearest splice junction, a biased nucleotide composition might be responsible for the overall increase in evolutionary rates with increasing distance from the splice site. To test this, we concatenated the alignments of all exons and for each distance value, we extracted a random sample from this concatenated alignment with the same sample size and nucleotide composition as the alignment sites for the respective distance from the splice junction. The overall evolutionary rates in the randomized samples are higher compared with the putative ESE motifs and comparable to the non-ESE sites, demonstrating that biased nucleotide composition cannot explain the lower evolutionary rates in putative ESE motifs. The magnitude of the difference between the ESE and non-ESE sequences in their rate of evolution in lincRNAs, with ESE evolving around 15% slower than nucleotide controlled null sequence, is the same as witnessed at 4-fold degenerate synonymous sites in protein-coding genes (Cáceres and Hurst 2014).

Although we employ experimentally confirmed ESEs, these are unlikely to correspond to all biologically meaningful ESEs. Nonetheless, selective pressure to maintain the experimentally defined set of ESE-motifs does not seem to be the only cause for this trend because non-ESE sequences show the same trend of increasing substitution rates with increasing distance (rho [ESE] = 0.554 and rho [non-ESE] = 0.729 for protein-coding genes; fig. 2, and for lincRNAs rho [ESE] = 0.298, rho [non-ESE] = 0.306; fig. 2). To consider more generally the role of splice-related constraint, we therefore also compare exon flanks with exon cores and with intronic cores. We presume any differences in rates to be owing to splice-related features.

#### Weak Constraint on lincRNAs Is Dominantly Owing to Selection in Exonic Flanks in Humans

We employed the ratio of the substitution rate in exons (*Ke*) over the substitution rate in introns (*Ki*) to scan the lincRNA alignments for signatures of purifying selection. For the interpretation of this ratio, introns are used as a proxy for background, possibly neutral, rate (Hoffman and Birney 2007; Resch et al. 2007). A *Ke/Ki* ratio < 1 (or < 0 after log-transformation) would thus be indicative of purifying selection. For protein-coding genes, there is evidence of higher selective constraints near exon–intron boundaries, both in the exonic and in the intronic regions flanking the splice junction (Chamary and Hurst 2004; Warnecke et al. 2008) and we therefore analyzed the regions in exon and intron cores and those flanking the splice junction separately (we do not analyze intron flanks). For each aligned gene in the protein-coding and the lincRNA data set, we concatenated 70 bp of exonic sequences flanking the splice junctions and calculated the number of substitutions in the concatenated exon flanks (*Kef*). We defined exon cores as the sequences enclosed by two exon flanks, concatenated them as well, and calculated their substitution rates (*Kec*). We compared those rates with the substitution rates in concatenated intron cores (*Kic*), defined as the intronic sequences without the 20 bp flanking the splice junction, as done previously (Warnecke et al. 2008). For this analysis, to reduce the impact of noisy short sequences, we excluded all genes for which the concatenated exon- and intron flanks and intron cores were shorter than 100 bp, which leaves us with 1,810 (53%) lincRNA genes.

**Fig. 3.**
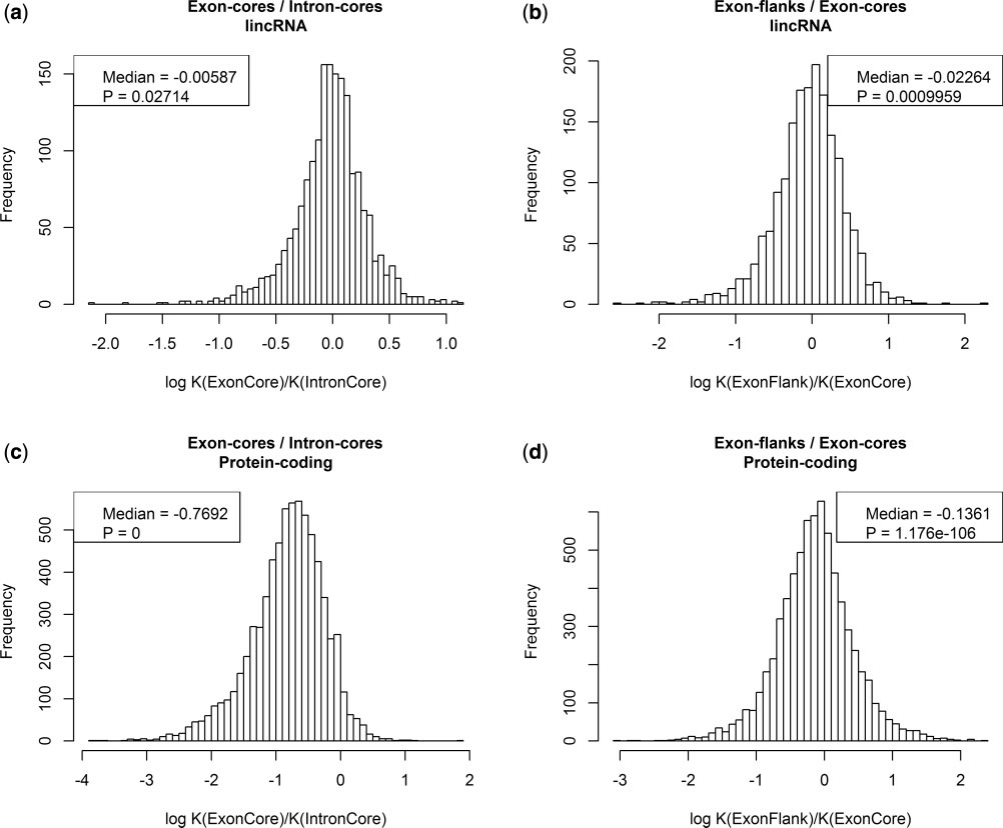
Exon cores and flanks evolve at different rates. The distributions of *Kec/Kic* and *Kef/Kec* values are shown for protein-coding genes and lincRNAs.

The logged (natural logarithm) distributions of *Ke/Ki* ratios for the alignments of lincRNAs and protein-coding genes are shown in figure 3*a* and *c*. The *Kec/Kic* distribution in protein-coding genes is, as expected, consistent with the majority of genes evolving under strong purifying selection (median log *Kec/Kic* = −0.769; fig. 3*c*). For the core exon and core intron regions of lincRNAs, we observe a similar but much weaker trend (median log *Ke/Ki* = −0.005, Wilcoxon test: *P* = 0.027; fig. 3*a*). This trend is still significant at the 0.05 level, but very weak compared with the same effect seen in protein-coding genes, which is consistent with earlier studies that found little evidence for purifying selection acting on lincRNA exons. The *Ke/Ki* ratio is slightly more pronounced when the entire set of lincRNAs instead of only the conservative subset is used (supplementary fig. S3, Supplementary Material online) which is not unexpected because the nonconservative set might contain some genes with protein-coding potential (see Materials and Methods for filtering with respect to coding potential).

We notice that with ESE sequence close to exon flanks evolving 15% slower than neighboring sequence, that this effect alone might explain all or nearly all constraint on ncRNAs. With about 27% of sequence near exon flanks (in the relevant sample), a density of ESE around 30% and 15% slower rate of evolution, assuming exonic non-ESE sequence evolves at about the same rate as introns, we predict that log *Ke/Ki* should be approximately log (1 − [0.27 × 0.3 × 0.15]) = −0.01. This is, if anything, greater than the proportional difference that is actually observed, suggesting that selection on ESEs may account for all of the reduced rate of evolution of exons compared with introns.

Assuming this to be the case, we would also expect a low rate of evolution at exon flanks to account for most of the difference between exon and intron. The exon flanks of protein-coding genes have previously been shown to evolve slower than the exon-core regions owing to the fact that splice-related motifs tend to be in the flanks (Parmley et al. 2007; Warnecke et al. 2008). As expected, we also observe this in our set of protein-coding genes (median log *Kef/Kec* = −0.136; fig. 3*d*). Importantly, a similar pattern can be observed for the lincRNA data set, where the exon flanks also evolve slower on average than the exon-core regions (fig. 3*b*). The difference between exon flanks and cores in lincRNAs is again not as pronounced as in the protein-coding data set but still highly significant (median log *Kef/Kec* = −0.022, Wilcoxon test: *P* = 0.0009). Overall then, these results support the view that the majority of selective constraint on lincRNAs is at exonic ends with little if any at exon cores. Put differently, if there are conserved motifs in exon cores (as seen if *Xist*), then they are rare. As we excluded genes where the concatenated exon flanks and cores or the concatenated intron cores were shorter than 100 nucleotides, these results do not necessarily reflect trends in short lincRNA genes.

These results replicate in part those of Chodroff et al. (2010) who noted a tendency for exon cores of lincRNAs to evolve faster than flanks. They suggest that this might not be owing to splice-related constraint but instead reflect transposable element insertion in cores. That in the flanks ESEs and non-ESE evolve at different rates (fig. 2) and that the net exonic rate can be predicted from knowing this constraint alone strongly argue in favor of splice-related constraint particular to the exon flanks.

#### Constraint on lincRNAs in Drosophila Is Stronger Than in Humans but Is Dominantly Not Owing to Selection in Exonic Flanks

We can ask whether the above result might be general. Recently, it has been reported that in *Drosophila* selection on ncRNAs is more intense than seen in humans (Young et al. 2012; Haerty and Ponting 2013). Does this mean that the difference in evolutionary rate between flanks and cores is all the more profound? To address this, we considered rates of evolution of ncRNAs as previously annotated comparing *D. melanogaster* and *D. yakuba*. Confirming the strong constraint in ncRNA exons we find that the log of the ratio of rate of evolution between exon core and intron core is −0.6 (Wilcoxon test: *P* = 4.8 × 10^−^^18^). Unexpectedly, we find that flanks evolve if anything faster than the cores (median ratio of log [flank/core] = 0.25, Wilcoxon tests: *P* = 1 × 10^−^^8^). We conclude that the stronger selection on flanks of ncRNAs is not universal.

### Causes of between-Gene Variation in Rates of Evolution

The above analyses indicate that ncRNAs use ESEs much as protein-coding genes do and that splice-related constraints explain the great majority of within-gene purifying selection. These results suggest a further issue. If splicing is so important in explaining intragene variation in rates of evolution, is it also the most important predictor of between-gene variation in rates of evolution? It is not trivially the case that this need be so. For protein-coding genes, a universal and highly significant negative correlation between gene expression and rate of protein evolution has repeatedly been observed (Pal et al. 2001; Drummond and Wilke 2008; Wolf et al. 2010). As this is effectively controlled for by considering intragene analyses, it could be that the causes of intragene variation are dwarfed by a feature, such as expression level, which only becomes important when considering intergene comparisons.

#### Partial Correlation Analysis Suggests ESE Density Is the Best Predictor of lincRNA Rate of Evolution

The above analysis suggests that ESEs and exon flanks impose major constraint on sequence evolution of lincRNAs. Indeed, the difference in the extent of constraint between the exon core and exon flank suggests that most constraint is splice related. This analysis, while controlled at a pairwise level, does not address the issue of how well splice-related constraints explain between-gene variations in evolutionary rate. How then do splice-related constraints compare with other putative predictors of evolutionary rate and how relatively important is each predictor when allowing for covariance with the others?

To this end, we carried out a partial correlation analysis using the *pcor* R script (Kim and Yi 2006). We considered three expression parameters (maximum expression rate, median expression rate and expression breadth, breadth being the proportion of tissues within which a gene is expressed), two splicing-related parameters (fraction of exon sequence in 70-bp windows flanking splice junctions [frac70] and the fraction of exonic sequence that matches known ESE motifs [ESE density]), folding stability and GC content. Normal and partial correlations are shown in table 1 (see also supplementary table S1, Supplementary Material online). In addition, for comparison, we consider the same parameters in their ability to predict rates of evolution of protein-coding genes.

**Table 1.** Normal and Partial Correlations with Evolutionary Rate (measured as Tamura–Kumar distance, see Materials and Methods) Using Spearman Correlation (for Pearson correlation, see supplementary table S1, Supplementary Material online).

	Normal	Partial
lincRNA		
Max. expression rate	−0.005	0.032
Med. expression rate	−0.025	−0.035
Exp. breadth	−0.038	−0.091**
RNA stability	0.048^†^	0.009
Frac70	−***0.051***^†^	−0.011
ESE density	−0.182***	−0.194***
GC	−0.058*	−0.102***
Protein coding		
Max. expression	−0.203***	−0.019
Med. expression	−0.339***	−0.063***
Exp. breadth	−0.369***	−0.189***
RNA stability	0.154***	0.028*
Frac70	−0.222***	−0.101***
ESE density	−0.29***	0.008
GC	0.313***	0.168***

Note.—Numbers highlighted in italic are significant after Bonferroni correction (at 5% level, raw *P* < 0.00357 with *N* = 14). Significance codes for *P* values prior to Bonferroni correction: ^†^*P* < 0.01; *P < 10^−3^; **P < 10^−6^; *** *P* < 10^−9^.

As regards the rate of evolution of lincRNAs, one parameter stands out. Out of all parameters we considered, the density of ESE motifs in exon sequences is the best predictor for evolutionary rates in lincRNAs, both in normal and in full partial correlation analyses. The other splicing-related parameter, the fraction of sequence within 70 bp of an exon junction (frac70), is however not significantly correlated with evolutionary rates of the lincRNAs.

For the protein-coding genes, the situation is somewhat different. Both the fractions of sequence within 70 bp of an exon boundary and ESE density are correlated with evolutionary rate in the normal correlation analyses, whereas ESE density is no longer significantly correlated in the partial correlation analyses. This seems to be an interaction effect with GC content because ESE density shows a significant partial correlation, comparable to the normal correlation, when GC content is removed from the set of controlled variables. The overall GC content of lincRNA exons is very low compared with the exons of protein-coding genes (median GC content = 0.309 and 0.515, respectively) which might explain why GC content masks the effect of ESE density on evolutionary rate in protein-coding genes but not in lincRNAs.

#### Expression Level Does Not Predict Evolutionary Rates of lincRNAs

A universal and highly significant negative correlation between gene expression and rate of protein evolution has repeatedly been observed (Pal et al. 2001; Drummond and Wilke 2008; Wolf et al. 2010). It has been proposed that the dominant underlying cause for this correlation is the cost imposed by protein misfolding, which is higher for highly expressed genes (Drummond and Wilke 2008; Yang et al. 2010). It has also been demonstrated however that the cost imposed by protein misfolding cannot be the only cause underlying the observed negative correlation, other possible mechanisms that underlie this correlation include the avoidance of protein misinteractions (Yang et al. 2012) and differential requirements for mRNA folding (Park et al. 2013).

If selective pressure to avoid protein misfolding (or indeed any protein related feature) is the cause of this correlation, a similar trend for lincRNAs should be absent, assuming that they are never translated into a protein product. However, Managadze et al. (2011) have shown that a weak but significant negative correlation between expression level and evolutionary rate is indeed observable in human and mouse lincRNA data sets. We tested the lincRNA data set produced by Cabili et al. to see whether we can reproduce these findings.

**Fig. 4.**
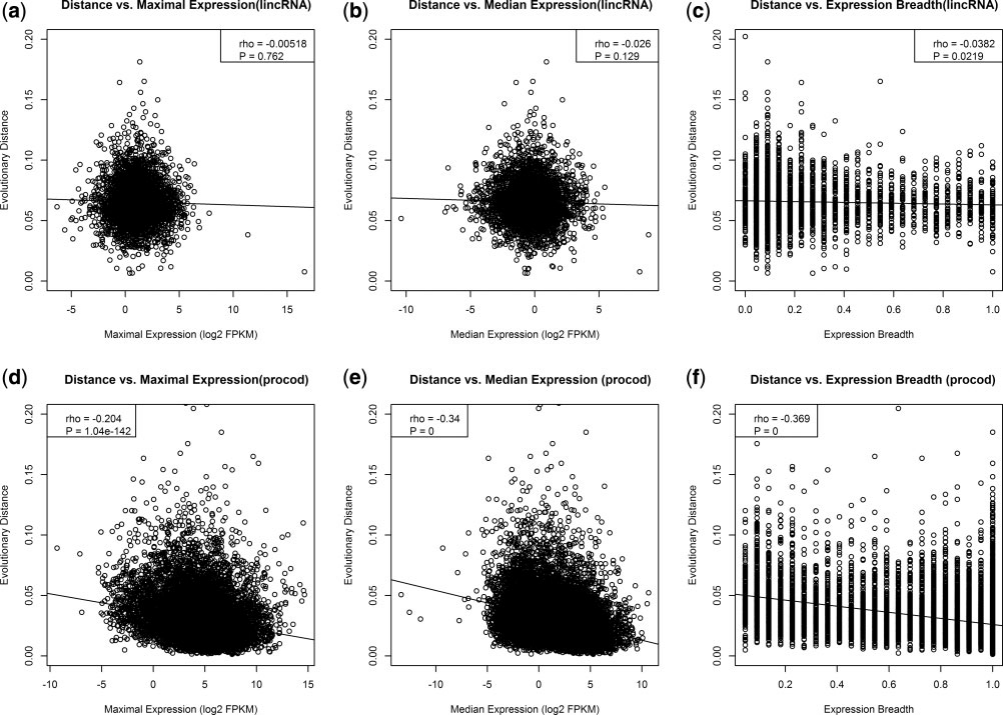
Correlation of expression parameters with evolutionary rates of lincRNAs and protein-coding genes. The evolutionary distance to the macaque homologue was plotted versus the values of maximum expression (*a*), median expression (*b*), expression breadth (*c*) for each lincRNA, and for protein coding genes (*d*-*f*).

For each lincRNA sequence, we plotted the evolutionary distance against the maximal and median expressions and the expression breadth of the respective lincRNA (fig. 4*a*–*c*). Surprisingly, given prior evidence (Managadze et al. 2011), we observe no significant correlation for either maximal expression (rho = −0.005, *P* = 0.76; fig. 4*a*) or median expression (rho = −0.025, *P* = 0.12; fig. 4*b*). This remains true after partial correlation. For expression breadth, we observe a weak negative correlation that is significant at the 0.05 level (rho = −0.038, *P* = 0.02; fig. 4*c*). This result is a little more robust on partial correlation analysis (table 1). Thus lincRNAs that are highly tissue-specific are, on average, less conserved between humans and macaques than those with a larger expression breadth.

The weak correlations between expression parameters and rate of evolution of lincRNAs contrast strikingly with what we find for protein-coding genes. For the protein-coding data set, maximum expression (rho = −0.204, *P* < 2.2 × 10^−^^16^; fig. 4*d*), median expression (rho = −0.34, *P* = 2.2 × 10^−^^16^; fig. 4*e*), and expression breadth (rho = −0.369, *P* = 2.2 × 10^−^^16^; fig. 4*f*) all show a highly significant negative correlation with expression, as expected. These results are diminished on partial correlation analysis but expression level and breadth remain predictors.

#### lincRNA Folding Stability Does Not Explain Evolutionary Rates

The possibility that RNA structure might be a determinant of protein rate of evolution has recently been proposed (Park et al. 2013). Given this it is relevant to ask whether the same may be perhaps an even more profound predictor for sequences where the RNA alone may be functionally relevant, that is, lincRNAs. We find that folding stability shows a very weak Spearman correlation with evolutionary rates of lincRNAs, but this effect vanishes when the other parameters are controlled for. We conclude that selection on folding strength does not explain the rate of evolution of long ncRNAs (lncRNAs).

Although one may question the ability of any method to correctly infer RNA stability (not least because they fail to acknowledge the presence of the exon-junction complex (EJC) on mature RNA), it is notable that this result contrasts with what is seen for protein-coding genes. In this instance, as previously reported (Park et al. 2013), folding stability shows a strong positive Spearman correlation with evolutionary rate (rho = 0.154, *P* < 2.2 × 10^−^^16^). We note an important word of caution, however, as this correlation is substantially reduced in the partial correlation analysis (partial rho = 0.028, *P* < 10^−^^3^). Moreover in a partial Pearson product–moment correlation the sign of the correlation shifts to being negative (supplementary table S1, Supplementary Material online). The strong correlation in the normal Spearman analysis (and that recently reported; Park et al. 2013) seems to be caused by an interaction effect with GC content and ESE density and the removal of those two parameters from the partial correlation analysis yields a partial correlation of comparable magnitude to the normal correlation (partial rho = 0.158, *P* < 2.2 × 10^−^^16^). GC content and folding stability are positively correlated and the negative correlation of folding stability and evolutionary rate thus seems to be caused by stable protein-coding RNAs having a higher GC content than average. Whether the GC content is high to ensure strong folding or whether strong folding is an incidental side consequence of GC content remains to be discovered.

#### Complex Correlations between lincRNA Folding Stability and Expression Parameters but No Relation with Evolutionary Rates

Although RNA stability does not predict evolutionary rates of evolution, it remains valid to ask whether stability might correlate with expression parameters. It has been proposed that many, if not most, lncRNA transcripts are highly unstable (Houseley and Tollervey 2009). However, genome-wide studies on lncRNA stability have revealed that lncRNA transcripts are not generally unstable, but rather show a wide range of stabilities that is on average lower, but still comparable to that of protein-coding mRNAs (Clark et al. 2012). As the stability of protein-coding RNAs is correlated with expression (Liebhaber 1997; Shabalina et al. 2006), we tested the lincRNA data set for the presence of a similar pattern.

**Fig. 5.**
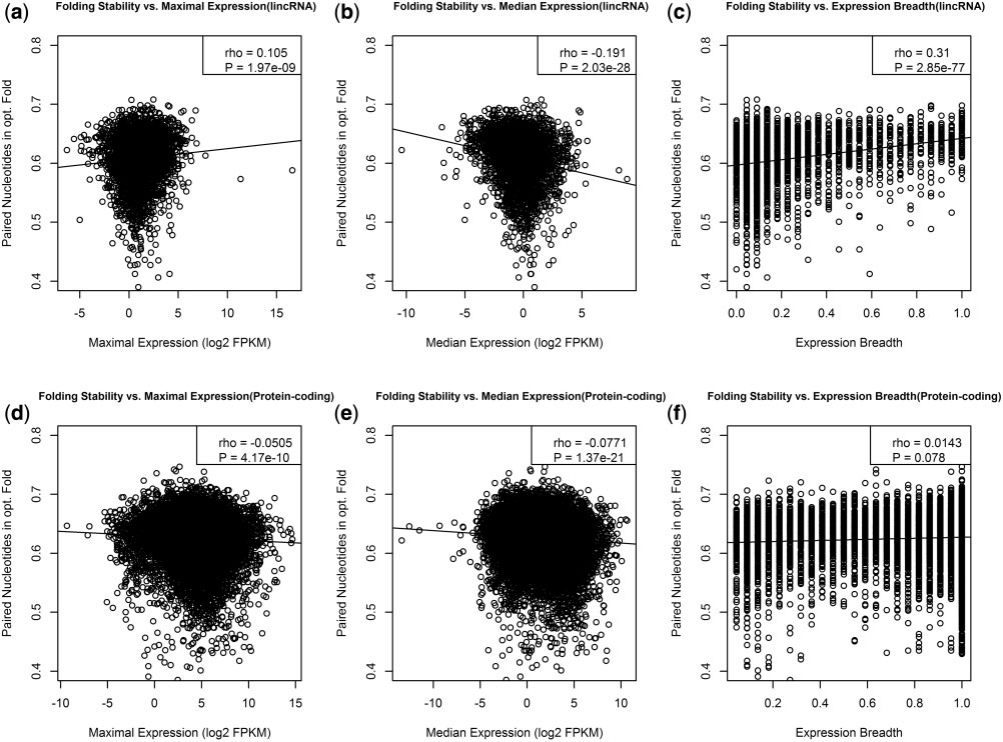
Folding stability and expression of lincRNAs. The folding stability, assessed as the fraction of paired nucleotides in the minimum energy fold, is plotted against maximum (*a*) and median expression (*b*) and expression breadth (*c*) comparable plots for protein coding genes are shown in *d*, *e*, and *f*.

We detected not only a significant positive correlation between folding stability and maximal expression level (rho = 0.105, *P* ∼ 10^−^^9^; fig. 5*a*) but also a highly significant negative correlation between folding stability and median expression (rho = −0.19, *P* < 10^−^^16^; fig. 5*b*) and a positive correlation with expression breadth (rho = 0.309, *P* < 10^−^^16^; fig. 5*c*). To see whether these trends are statistically independent from each other, we constructed a linear regression model to predict RNA stability based on all three expression parameters and conducted an analysis of variance. There is a significant three-way interaction between maximum expression, median expression, and expression breadth (*F*-test: *P* ∼ 10^−^^5^). These trends suggest that stable lincRNAs are associated with a high maximum expression and are expressed in several tissues, but are highly expressed in few or only one of these tissues and thus also have a low median expression.

For the protein-coding genes in our data set, we observe negative correlations between folding stability and both maximal expression (rho = −0.05, *P* < 10^−^^9^; fig. 5*d*) and median expression (rho = −0.07, *P* < 10^−^^16^; fig. 5*e*) but no significant correlation with expression breadth (fig. 5*f*). This is perhaps surprising as expression breadth is the strongest predictor of protein evolutionary rates.

#### Differential Sampling with Respect to Expression Level Explains Differences between Analyses

The above analyses have thrown up two possibly surprising results: Splice-related features are centrally important for predicting between-gene variation in rates of evolution and expression level appears not to be an important predictor. The latter result is doubly surprising given how important expression level is for predicting protein rates of evolution and because Managadze et al. (2011) had previously reported a coupling between evolutionary rate and expression rate. There may be several reasons why this correlation is not apparent in the lincRNA data set produced by Cabili et al. The data set used in the study by Managadze et al. was based on lincRNA data from the NRED database (Dinger et al. 2009) and has a smaller sample size compared with the data set we used in this study. Using a gap threshold of 15% (to match that of Managadze et al., see Materials and Methods) we are left with 3,592 lincRNAs compared with 519 human and 2,013 mouse lincRNAs in the study by Managadze et al. This may itself have some influence, as if we reduce our sample size to 1,500 transcripts we recover a negative correlation at least as extreme as that seen in Managadze et al.’s study 18% of the time.

However, the more important reason for the discrepancy appears to be that the data produced by Cabili et al. is based on deeper transcriptome sequencing that is less biased toward highly expressed lincRNA genes. We tested this hypothesis by analyzing the correlation between expression level and evolutionary rate using only the 50% of lincRNAs that show the highest maximum expression level. For this data set, we do observe a weak but significant negative correlation (supplementary fig. S4*A–C*, Supplementary Material online, *P* values for all correlations < 0.05). Indeed, when we repeatedly subsample from only the more highly expressed gene set we recover a negative correlation at least as extreme as that seen in Managadze et al.’s (2011) study 98% of the time. We obtained the lincRNA data set used in the study by Managadze et al. (2011) and can reproduce their results using this data set.

These results suggest that the correlation observed before between expression level and rate of evolution of lincRNAs (Managadze et al. 2011) is dependent on limited sampling. It might be that the more in-depth analysis of Cabili et al. (2011) is more noisy, especially for lowly expressed transcripts, and that this extra noise led to the removal of the correlation. Alternatively, there may not be a monotonic relationship between expression level and rate, in which case sampling only the more highly expressed transcripts could enable detection of a strong trend unique to the highly expressed genes. Alternatively, the prior result may simply be an artifact of limited sampling. As we cannot discriminate between these alternatives, we suggest that the evidence against the misfolding hypothesis on the basis of correlation between expression level on ncRNA evolution (Managadze et al. 2011) be considered provisional. The possibly stronger evidence (although this is relative) appears to derive from a weak correlation between expression breadth and rate of evolution.

## Discussion and Further Results

Human lincRNAs have been shown to be almost as poorly conserved as other intergenic sequences and even highly functional lincRNAs such as *Xist* only contain few short stretches that are well conserved (Pang et al. 2006). However, many lincRNA loci have conserved promoter sequences (Carninci et al. 2005) and conserved splice sites (Ponjavic et al. 2007). The 5′- and 3′-splice sites alone are usually not sufficient to maintain proper intron excision (Lim and Burge 2001) without the additional presence of ESE motifs near the splice junction (Wang et al. 2005). In protein-coding genes, purifying selection is acting to maintain these motifs and we thus hypothesized that, given the presence of conserved splice sites, a similar trend might be observable for lincRNAs. Consistent with this hypothesis, we do observe that the exon-flank regions of lincRNAs evolve slower than their exon-core regions. This trend is weaker than the one observed in protein-coding genes, but qualitatively similar and highly statistically significant. We further showed that the putative ESE motifs within exon flanks evolve significantly slower than the sites which do not correspond to known ESEs, indicating that purifying selection to maintain ESE motifs is at least partly responsible for the slower evolution in exon flanks compared with core regions.

One possible explanation for the difference in evolutionary rate between ESE and non-ESE at exon flanks (fig. 2) is that our data set of lincRNAs is contaminated with protein-coding genes and these and these alone employ ESEs (or employ them much more often). Note that if both protein-coding genes and noncoding genes both employ ESEs at similar densities (which we show they do—fig. 1*a* and *b*), then the difference in rate between ESE and non-ESE cannot be explained as a contamination artifact. On a priori grounds, it is indeed hard to see how an SR protein might distinguish an ESE in a protein-coding immature transcript from the same ESE in a lincRNA immature transcript. The artifact explanation we suggest is unparsimonious for numerous reasons. First, all of our results strongly argue against a large contamination issue: The rate of evolution is extremely high on average in our lincRNAs and we do not recover the strongest protein-related correlations, such as with expression level and RNA stability. Perhaps more directly, if we split our internal exons into those with at least one stop codon in every frame (very unlikely to be protein coding) and all others and repeat the analysis of ESE and non-ESE rates, we observe that the two partitions of the data are nearly identical in absolute rates and the difference between ESE and non-ESE (supplementary fig. S5, Supplementary Material online). Indeed, the difference between ESE and non-ESE in the set with stops in all frames is approximately 15% as it is for the data set en mass and for 4-fold degenerate sites in protein-coding exons (Cáceres and Hurst 2014). We conclude that our results are not affected by contamination from protein-coding sequence.

Given the evidence for purifying selection on ESEs we might expect to see some genetic diseases associated with splicing defects in lincRNAs owing to single nucleotide polymorphisms close to but not at the splice junctions. The evidence for selection on ESE refutes the hypothesis that lincRNAs are the all the product of junk transcription. That the majority of the selection on lincRNAs is on splicing presents a new paradox, why it is that selection acts on the splicing process. In principle there might be at least three classes of explanation, which we term the product hypothesis, the error-proofing hypothesis, and the process hypothesis.

### Why Is Most Selection on Human lincRNAs Splice Related?

#### Absence of Constraint in Exon Cores Does Not Refute the Product Hypothesis

The product hypothesis proposes that the product of transcription and splicing is important and the precise exonic structure of the mature ncRNA relevant to this function (as with most protein-coding genes). Our finding that exon cores evolve at rates very similar to those of flanking introns provides little or no support for the idea that functionality of the ncRNA product impacts evidently on sequence conservation. This does not, however, refute the product hypothesis for several reasons. First, some well-described lincRNAs have known functions (e.g., Xist) but apparently little or no sequence conservation (Pang et al. 2006). Further, were our lincRNAs to contain very small subset of sites under strong purifying selection owing to selection on the operation of the RNA, we would almost certainly be unable to detect it with our metrics, the sites being too rare and hence diluted. In addition, conservation of function may be reflected not in conservation of nucleotide sequence but in tolerated indel events.

One way to rationalize the apparent rare selection on nucleotide sequence is that ncRNA might be under selection to enable strong structure, which imposes only weak selection on the primary sequence. This hypothesis would also be consistent with the observations that many lincRNAs with distinct sequences are able to bind the same protein complex (Guttman et al. 2011; Khalil and Rinn 2011) and that the rates of insertions and deletions, which would be much more disruptive to the secondary structure than point mutations, are reduced in lincRNAs (Ponjavic et al. 2007). Were this the case, however, we might expect that lincRNAs that are more stable might evolve slower to preserve that structure. Our data, however, find no support for the view that lincRNA structure is under selection, or at least that any selection on structure is operating uniformly in the same direction (e.g., to always increase stability). This contrasts with the picture for protein-coding genes and with prior claims for ncRNA (Managadze et al. 2011). Selection against indels may well also disrupt splicing, potentially explaining this prior result. In sum, given evidence of function in the absence of sequence conservation, we cannot eliminate the hypothesis that lincRNAs have a direct function, we just find little or no evidence to support it from the mode of sequence evolution in humans, although the fly data are compatible with such a model.

#### No Strong Evidence for the Nonsense-Mediated Decay Error-Proofing Hypothesis

Another possibility is that the selection on splicing may be part of an error-control mechanism. Splicing commonly results in the mature transcript being bound with the EJC in proximity to the exon–exon junction (Le Hir et al. 2001). The complex is known to mediate the effect of splicing on mRNA expression levels (Wiegand et al. 2003) and so might be directly beneficial were the lincRNA functional (i.e., the product hypothesis above). However, many of the effects of the EJC may well be undesirable for ncRNAs: The EJC acts to promote export from the nucleus, enable polyadenylation, and enhance translation (Le Hir et al. 2001; Wiegand et al. 2003). In addition, however, in mammals the EJC is also necessary for the initiation of NMD (Le Hir et al. 2001; Isken et al. 2008). Might the selection on splicing be to enable the EJC to be attached to initiate NMD should a ribosome inappropriately bind an ncRNA? Binding of ribosomes to ncRNA has been described (Wilson and Masel 2011), but whether this reflects improperly annotated coding genes or accidental ribosome initiation in unclear.

At first sight this is a possibly attractive explanation, not least because it is consistent with the apparent-reduced constraint in exon flanks compared with cores in *Drosophila*, as flies do not employ the EJC to initiate NMD (Brogna and Wen 2009). The hypothesis also fits with the notion that many otherwise paradoxical features of gene and genome evolution are error-correcting or error-proofing (Warnecke and Hurst 2011). However, the hypothesis has at least one major problem. Although a polyA tail is required for NMD activation (Brogna and Wen 2009), many ncRNAs are possibly not polyadenylated. Indeed, the great majority of the transcripts that can be detected uniquely in protocols that do not require polyA tail tagging, compared with methods that require such tagging, are lncRNAs (Cui et al. 2010).

We can in addition ask whether we can find a trace of selection for NMD triggering on the ncRNA sequences. Stop codons less than about 50 bp upstream of the terminal exon–intron junction are thought to be invisible to the activity of NMD (Zhang et al. 1998), what we term the NMD shadow. This provides grounds for potentially instructive tests. If introns are there to trigger NMD, then sequence prior to this 50-bp window might be expected to have a higher frequency of stops (in any frame). To address this, then we considered instances of lncRNAs where the last but one exon was more than 100 bp. We then considered the 50 bp at the 3′-end of this exon and the 50 bp at the 5′-end of the same exon. We then compare stop codon frequency in the 5′- and 3′-end in a paired fashion. This method allows us to control for the amount of sequence analyzed per exon, the proximity to an exon junction (given that these are expected to be purine loaded owing to the presence of ESEs), isochore level nucleotide content, and the possibility that the last but one exon may actually be protein coding. A small and nonsignificant minority (47.5%) of last but one exons have more stops at the 5′-end than the 3′-end (binomial test: *P* = 0.31). On average, each 50-bp exon end has about 0.6–0.7 stop codons in each reading frame. We thus see no evidence that stops are enriched outside of the NMD 50-bp shadow.

One might object that this test fails to recognize the possibility that a stop may have occurred prior to the last but one exon and only one stop is required (per possible frame). To consider this, then we consider the class of ncRNAs with just two exons and consider the sequence −100 to −51 prior to the single exon junction in the first exon and compare this to sequence −50 to the 3′-end of exon (the NMD shadow). As before the stop codon frequency is no different in the two (in 50.03% of cases the first 50 bp has the higher stop codon frequency). Of all two exon ncRNAs 20% have no stop codon outside of the final 50 nucleotides and 55% have fewer than three, meaning that at least one prospective reading frame is NMD unprotected. Were there selection for stops outside of the NMD shadow this should be most apparent in those first exons longer than 50 bp but still relatively short. Of those first exons that have more than 50 bp of sequence but less than 101 bp, 45% have no stop codons outside of the NMD shadow. Randomizing the same 5′-sequence we predict that around 41% would lack a stop codon in any frame by chance alone suggesting, if anything, that the real sequence is slightly diminished for stops. Ninety-one percent of the 5′-sequences have fewer than three stop codons meaning that at least one frame of reading is NMD unprotected. Eighty-six percent of random sequence is expected to have fewer than three, again suggesting no enrichment of stop codons to initiate NMD. We can more generally ask about stop codon density in the 5′-exon of two exon genes. If stops are there to trap ribosomes, then we would expect a higher density in small first exons as these would be under particular pressure to encode them, longer first exons likely having a stop codon by chance. However, stop codon density is unrelated to exon length, with no hint of the expected negative correlation (rho = 0.14, *P* = 0.51). In sum, we find no good evidence that selection is enriching these exons for stop codons to trigger NMD.

#### The Process Hypothesis: Intron Density Is Associated with Chromatin and Gene Activity

The final possibility is that it is the process of splicing that is important. Implicit in the process argument, and contrary to the product hypothesis, is the notion that after the splicing event the RNA could be destroyed instantaneously with no negative consequence. Although it is unclear why the process might be relevant, we note that recent evidence suggests that the splicing process is somehow coupled with epigenetic marks on the DNA (Adam-Hall and Georgel 2011; Luco et al. 2011). This can mean both that the epigenetic status of the DNA can affect the process of splicing and, more importantly in this context, that the splicing process can modify the underlying DNA (Hnilicova and Stanek 2011). Evidence exists for both directions of interaction (Hnilicova and Stanek 2011). Mechanistically it is unclear how this operates but four chromatin adaptor proteins (including the chromatin remodeler CHD1 [Sims et al. 2007]) are recognized that permit coupling between splicing factors and histone posttranslational modifications (Adam-Hall and Georgel 2011). Similarly, the SWI/SNF chromatin remodeling factors are known to interact with many components of the spliceosome (Adam-Hall and Georgel 2011). We note that a potential role for introns in modulating the chromatin of the underlying gene has relevance for explaining why mammalian transgenes typically require introns for efficient expression. As this effect is mediated, at least in part, by the recruitment of the EJC, rather than the presence of an intronic sequence per se (Wiegand et al. 2003), interaction between the EJC and any of the above splice/chromatin modifiers would provide a mechanistic rationale.

Given that ncRNAs are thought to play a role in chromatin modulation (Mercer and Mattick 2013) (although splicing is not always necessary [Beckedorff et al. 2013]) this suggests a hypothesis that is, to the best of our knowledge, novel. Might the expression and splicing on lincRNAs be a mechanism to alter the epigenetic landscape of the underlying DNA? If it does, then might this simply be a mechanism to control expression of the lincRNA or might it have knock on consequences for flanking genes? In yeast and mammals, for example, the expression of one gene causes a time-lagged ripple of gene activation of neighbors associated with spreading altered chromatin (Ebisuya et al. 2008). NcRNAs are well known to have *cis*-effects on genes in their vicinity (Pauler et al. 2012), so this possibility is not without precedent. Here then we ask two questions. First, is there evidence consistent with the possibility that lincRNAs affect, through splicing, underlying chromatin? Second, is there evidence consistent with the possibility that the activity and splicing of lincRNAs impact on the chromatin and expression of neighbors? Note that expression alone might have effects on chromatin even in the absence of splicing (as in yeast), such a model can also apply to expression of lincRNAs without introns. Our hypothesis is that splicing can bolster such an effect.

This hypothesis, like the NMD hypothesis, can potentially explain why constraint is not so evident in exonic flanks in *Drosophila*. Although chromatin modifiers can also be splice modifiers in *Drosophila* (Hnilicova and Stanek 2011), in flies ESE density is thought to be relatively low as introns are short and exons typically have strong splice sites (Warnecke et al. 2008). Humans, in contrast, have much longer introns and quite often weaker splice sites, both of which predict higher ESE density (Dewey et al. 2006). Thus information in the flanks is thought to be of lesser importance in *Drosophila* than it is in humans for the specification of splice location and, while detectable, the impact on codon usage and rates of evolution at exonic flanks of selection for ESEs is marginal in protein-coding genes (Warnecke and Hurst 2007).

##### Intron-Rich Active lincRNAs Are Enriched in CHD1.

To test the first hypothesis, we assessed 1) whether actively transcribed lincRNAs are enriched in CHD1-binding sites compared with inactive lincRNAs and 2) whether the density of CHD1 is correlated with the intron density (and hence the amount of splicing per base pair of a gene). This analysis is limited to the cell lines H1-HESC, from human embryonic stem cells, and K562, a leukemia cell line, as those are the only cell lines for which CHD1 modifications are available in the ENCODE data set. lincRNA expression status and expression of neighbors we derive from the same two cell types. Note that the genes considered active or inactive in the two cells in these analyses are specific to each cell and the CHD1 measure is similarly specific to each cell type. Thus, the two cell types are independent tests of the same hypothesis.

We find that active lincRNAs are more dense in CHD1 on the DNA containing the gene compared with the transcriptionally inactive lincRNAs (Mann–Whitney *U* test, two tailed, *P* = 3.9 × 10^−^^9^ in H1 and *P* = 4.8 × 10^−^^9^ in K562). Concerned that this statistic may be misled by large number of sequences with no CHD1 binding, we repeated the analysis using a Monte Carlo simulation (see Materials and Methods) which may be more robust to the data structure. The results remain robust (from simulation: *P <*< 10^−^^4^).

**Fig. 6.**
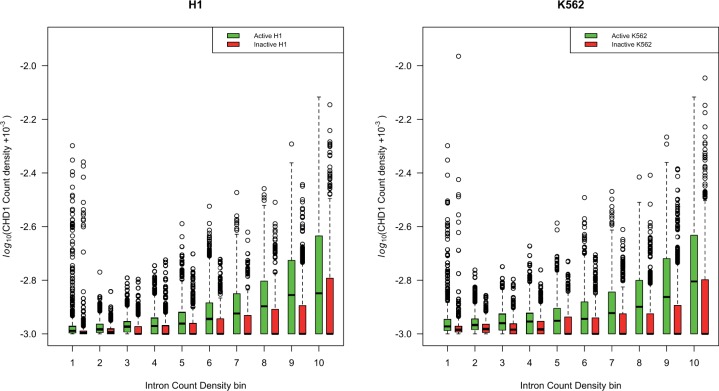
CHD1 density within lincRNAs is higher in active intron rich genes. Here, for each gene, we consider the number of CHD1 peaks (as specified by ENCODE) per unit base pair of each gene and compare this with the number of introns per unit base pair of gene length (in both cases we employ the length of the unspliced gene). We consider those lincRNAs that are transcriptionally active or inactive in each cell type separately. As can be seen, active genes have higher CHD1 density the more introns they have. For H1 active, rho = 0.23, *P < *2.2 × 10^−16^, for K562 rho = 0.16, *P < *2.2 × 10^−16^. For the inactives, the inverse is seen the effect being greatly owing to the great number of intron rich genes without any CHD1: For H1 inactive, rho = −0.11, *P < *5.2 × 10^−15^, for K562 rho = −0.19, *P < *2.2 × 10^−16^. Concerned that there were many tied values we examined the latter result using the Goodmans Kruskall gamma test, this being more robust to tied values. Results are unaffected (for H1 active, gamma = 0.2048, H1 inactive gamma = −0.0863, K562 active gamma = 0.1353, and K562 inactive gamma = −0.1382; all *P*’s < 0.001 from 1,000 simulations). Note that the genes considered active or inactive in the two cells are specific to each cell and the CHD1 measure is similarly specific to each cell type. Thus, the two cell types are independent tests of the same hypothesis. Considering CHD1 coverage (i.e., proportion of gene covered by at least one CHD1 span) does not affect conclusions: H1 active, rho = 0.1, *P < *10^−12^, K562 active rho = 0.08, *P* < 10^−8^, inactives: H1 rho = −0.15, *P < *2.2 × 10^−16^, K562 rho = −0.23, *P < *2.2 × 10^−16^. Results are again robust to application of Goodmans Kruskal gamma (H1 active gamma = 0.0865, K562 active gamma = 0.0654 and H1 inactive gamma = −0.142 and K562 inactive gamma = −0.1834 and all *P* < 0.001, from 1,000 simulations).

**Fig. 7.**
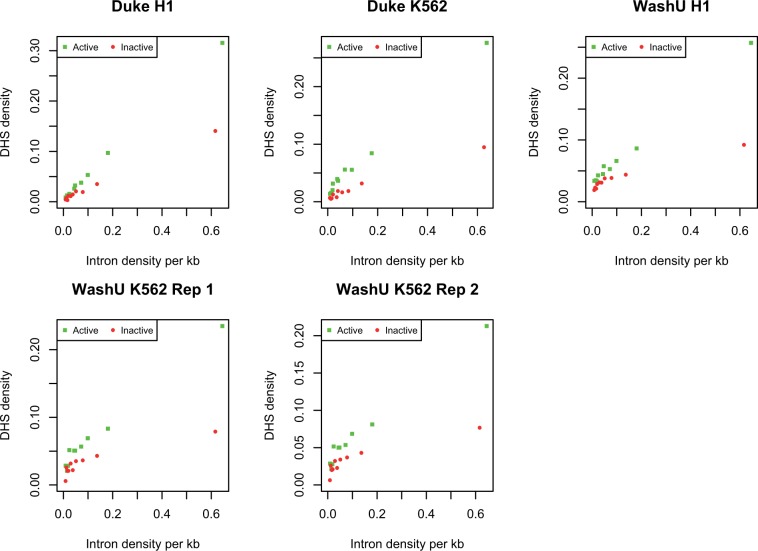
DHS density as a function of intron density for active and inactive genes. As within the WashU data set DHS density is rather low (such that most short genes have no DHS peak within the gene), we here analyze the data in manner designed to avoid the inherent stochasticity this induces. First, we rank all genes by total gene length (including introns). We then divide the data into bins of equal total gene size. With ten bins, the first bin contains the longest genes whose total length in approximately 1/10 the total gene length. Thus, each bin has different numbers of genes but an equal amount of total sampled DNA. We then calculate for each bin the total number of introns to derive the number of introns per kilobase of sequence. We also consider the total number of DHS peaks and calculate the number of these per kb. All correlations are significant at *P* < 0.0002 (Spearman). In all incidences, the mean DHS density is higher in the actives than the inactives (paired *t*-test, *P < *0.05).

In addition, we can ask whether the CHD1 density on a ncRNA is predicted by the density of introns. As can be seen (fig. 6), active genes have higher CHD1 density the more introns they have (H1; rho = 0.23, *P < *2.2 × 10^−^^16^, for K562 rho = 0.16, *P < *2.2 × 10^−^^16^). For the inactives, the inverse is seen, the effect being greatly owing to the great number of intron-rich genes without any CHD1 (H1 inactive, rho = −0.11, *P < *5.2 × 10^−^^15^, for K562 rho = −0.19, *P < *2.2 × 10^−^^16^). This might suggest active purging of CHD1 from inactive genes. Considering CHD1 coverage (i.e., proportion of gene covered by at least one CHD1 span) does not affect conclusions: H1 active, rho = 0.1, *P < *10^−^^12^, K562 active rho = 0.08, *P* < 10^−^^8^, inactives: H1 rho = −0.15, *P < *2.2 × 10^−^^16^, K562 rho = −0.23, *P < *2.2 × 10^−^^16^. These tests are robust to application of Goodman–Kruskall gamma test, a test more robust to tied values (see fig. 6). In turn we can ask whether CHD1 occupancy correlates with the extent of open chromatin within the genes in question, as assayed by the density of DNAase Hypersensity Sites (DHS). As expected, active genes have higher DHS than inactive ones and the extent of DHS correlates positively with intron density (fig. 7). There is a strong positive correlation between CHD1 occupancy and DHS occupancy in both cell types (table 2).

**Table 2.** Correlation between Intragenic DHS Density and CHD1 Coverage Density Occupancy within Active Genes.

Source of DHS Data	H1	K562
Duke	*P* < 2.2 E-16	*P* < 2.2 E-16
	rho = 0.43	rho = 0.51
WashU	*P* < 2.2 E-16	Rep1:
	rho = 0.44	*P* < 2.2 E-16
		rho = 0.30
		Rep2:
		*P* < 2.2 E-16
		rho = 0.31

Note.—lincRNA data from Derrien et al. (2012). WashU DHS data provide two replicates for K562. We analyze both separately.

These findings are consistent with the chromatin modification/splicing hypothesis, in which splicing recruits CHD1 to the underlying sequence which in turn acts to maintain or force opening of chromatin. Moreover, consistent with the notion that splicing enables the focal gene to remain open and active we find that the intron density in both cell lines is higher for active genes than for inactive ones (Mann–Whitney *U* test: H1, *P* = 0.0003, K562 *P* = 0.04).

##### Intron Density of lincRNA Predicts Local DHS Density and Expression of Neighbors.

Although the above evidence is consistent with the hypothesis that splicing of lincRNAs mediates recruitment of CHD1 to the underlying DNA, it provides no evidence that this has consequences for the neighboring genes. It may simply be the case that CHD1 recruitment aids the maintained expression of the focal lincRNA (for whatever reason) or indeed, that CHD1 recruitment is an incidental occurrence, a necessary consequence of splicing. We can then also ask whether active lincRNAs define a broader domain of open chromatin and a domain of increased gene activation, as supposed by the ripple hypothesis (Ebisuya et al. 2008). To this end we ask about activity in the domains flanking the focal genes, both in terms of chromatin and gene activity. As in humans the ripple effect is thought to extend approximately 100 kb (Ebisuya et al. 2008), this defines the span that we examine.

**Fig. 8.**
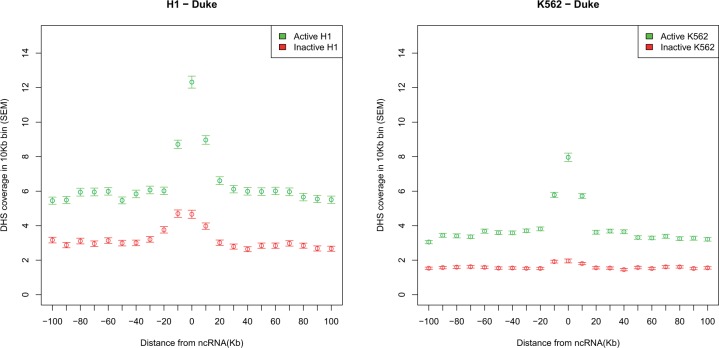
The DHS density in sites flanking active and inactive lncRNAs.

To analyze the chromatin state, we examine the density of DHS in spans around the focal active or inactive lincRNAs. We calculated the DHS density in independent 10-kb windows either side of active and inactive lincRNA and then compare the density, at a given distance between the active and inactive ones. As can be seen the DHS density is highest in the immediate vicinity of active loci in both cell types (fig. 8 and supplementary fig. S6, Supplementary Material online). It is striking that active lincRNAs appear to be at the position of maximal chromatin opening. This is what would be expected were activity of the lincRNA causing a rippling/spreading opening of chromatin.

If the chromatin splice model is correct and enables spreading of open chromatin, then we might expect that the local DHS density is correlated both with intronic density and with activity of the focal gene. To examine this, we consider the 50-kb blocks either side of the focal gene and take the average DHS density. We then ask whether that density is correlated with the intron density of the focal gene. We find that it is both for active and inactive genes (table 3; supplementary fig. S7, Supplementary Material online). Similarly, the CHD1 density in the focal gene predicts DHS coverage in the flanking sequence (table 4), this effect being either about the same magnitude as in the inactives or much more profound when the focal gene is active, depending on the data set. To ask then whether the inactives and actives differ in the local DHS density controlling for gene intron content, we perform a loess regression and compare the residuals for the actives and inactives. We consider numerous alternative kernels for the loess to consider the consequence of different smoothing parameters. In all cases, the actives have a higher DHS density in their vicinity than the inactives (supplementary table S2, Supplementary Material online). We conclude that high intron density, high CHD1 occupancy, and gene activity of the focal lincRNAs all predict higher DHS levels in the neighborhood of the active gene, consistent with a spreading chromatin model.

**Table 3.** Spearman Correlation between the Focal lincRNA Gene’s Intron Count Density per Kilobase and DHS Coverage per Kilobase in ± 50 kb Flanks.

Flanking Data (±50 kb)	Active Rho	Active *P*	Inactive Rho	Inactive *P*
Duke H1	0.264	2.34E-78	0.219	1.82E-50
Duke K562	0.239	1.11E-63	0.223	3.38E-52
WashU H1	0.183	5.49E-38	0.132	6.62E-19
WashU K562 Rep1	0.190	1.49E-40	0.191	1.31E-40
WashU K562 Rep2	0.146	4.12E-23	0.142	7.16E-22

**Table 4.** Spearman Correlation between the Focal lincRNA Gene’s CHD1 Density per Kilobase and DHS Coverage per Kilobase in ±50 kb Flanks.

Flanking Data (±50 kb)	Active Rho	Active *P*	Inactive Rho	Inactive *P*
Duke H1	0.334	1.72E-127	0.303	3.84E-97
Duke K562	0.465	1.02E-258	0.264	5.14E-74
WashU H1	0.455	2.09E-247	0.369	6.16E-147
WashU K562 Rep1	0.528	0	0.529	0
WashU K562 Rep2	0.286	9.65E-87	0.286	5.69E-87

**Fig. 9.**
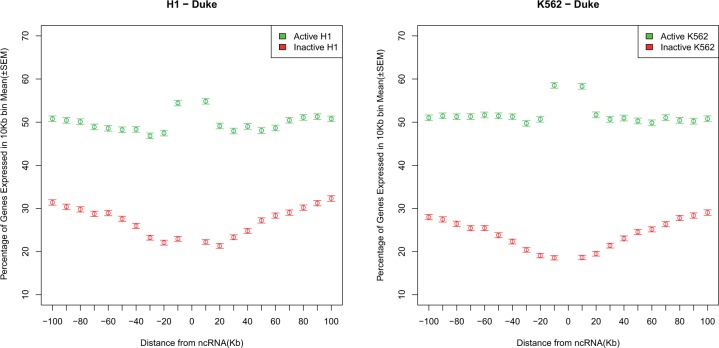
Gene expression in the vicinity of active and inactive lincRNAs.

We can in addition ask whether this open chromatin has any functional correlates. We might, for example, imagine that upregulation of a lincRNA with a high intron density modifies local chromatin and enables genes in the vicinity to be expressed by spreading of chromatin. Consistent with this, the neighbors of active lincRNAs are themselves especially active. Just as active genes sit at local DHS peaks, so too active lincRNAs sit at the centre of peaks of expression (fig. 9 and supplementary fig. S8, Supplementary Material online). The DHS and the expression modulation peaks extend approximately the same distance. As expected given this model, intron-rich and intron-poor lincRNAs have differing gene activity in their vicinity (correlation between intron density and percentage of genes expressed, for H1 and K562 active rho = 0.08, *P < *1 × 10^−^^8^) (supplementary fig. S9, Supplementary Material online). This correlation is slightly weaker when the focal gene is inactive (for both rho = 0.07, *P* < 10^−^^6^). Similarly the focal gene’s CHD density positively correlates with the expression of neighbors (H1 actives, rho = 0.19, *P* = 1.2 × 10^−^^43^; K562 actives rho = 0.18, *P =* 6.7 × 10^−^^35^). Again controlling for intron density, using the loess method, we find that active lincRNAs have higher gene expression in their vicinity than inactive ones (supplementary table S3, Supplementary Material online).

The above evidence is consistent with the model that transcription and splicing of lincRNAs modulate chromatin of the underlying gene body which can in turn have a spreading effect, modulating expression of neighbors.

### Alternative Models and Interpretations

Above we discussed three possibilities but this catalogue of possible explanations is by no means exhaustive. It has, for example, been demonstrated that ncRNAs can act as long-range *cis*-silencers by transcriptional interference, and could thus be regulatory active without the mature RNA being involved in the process (Pauler et al. 2012). This would be a further manifestation of the process argument, although why this process requires splicing is unclear. There might in turn be selection for the correct placement of the EJC, for reasons other than the initiation of NMD. EJCs are, for example, thought to regulate RNA localization (Giorgi and Moore 2007). Given that EJC placement is not now thought to be constitutive (Sauliere et al. 2010), it will be informative to know whether lincRNAs are unusual in their ability to attract these complexes.

Our chromatin model results are consistent with a model in which splicing of lincRNAs recruits CHD1 (and related splice associated chromatin modifiers) to transcriptionally active DNA, and this in turn enables both the chromatin within the focal gene to remain open and for there to be some spreading away from the focal active gene which is permissive for expression of neighbors. The same model, we note, also suggests a novel hypothesis for the positive correlation between intron density and expression breadth of protein-coding genes (Parmley et al. 2007) on the one hand, and the tendency for house-keeping genes to genomically cluster (Lercher et al. 2002). Broadly expressed (housekeeping) genes may be selectively favored to have absolutely more introns to enable a self-reinforcing open chromatin (N.B. intron density is higher in active genes). This would be mediated by splicing increasing the chances of recruiting CHD1 (and similar splice/chromatin modifiers) to the local DNA, which increases the chances of keeping chromatin open, enabling a higher likelihood of further transcription of the neighboring broadly expressed genes.

However while consistent with the model, our results are also consistent with alternative models. Notably, if for some other reason intron-rich genes tend to reside in domains of high gene activity then it is possible that the lincRNA has expression passively dependent on the local DHS/expression environment, much as transgenes adopt the expression profile of neighbors (Gierman et al. 2007). Consistent with this model highly and broadly expressed genes cluster in mammalian genomes (Caron et al. 2001; Lercher et al. 2002). Similarly, GC rich isochores tend to be domains of small introns and hence a higher intron density measured as introns per base pair of full gene. Note, however, in this model, given the evidence of local transgene adoption of expression profiles (Gierman et al. 2007), there is still a need to evoke the notion that local gene expression influences genes in the vicinity. Indeed, to disallow the possibility that lincRNAs do not affect their neighborhood, one would have to make a special case as to why one class of gene (protein coding) might affect neighbors but another (lincRNAs) do not. Why in this passive expression model intron dimensions of the focal lincRNA covary with local expression level is unclear, but could reflect a local mutational bias toward deletions. Indeed, intron size and intergene distance tend to covary (Urrutia and Hurst 2003).

The required experiment to distinguish these two explanations would be the introduction of a lincRNA with and without introns and ask whether the intron containing one affects the local DHS and expression of neighbors more than the same insertion when lacking the intron. It would also be helpful to know whether transcription rate of a lincRNA, with or without introns, might predict the extent of any upregulation of neighbors. Should it prove to be the case that intron-bearing genes modulate the expression of their neighbors, this would have consequences for the assessment of the safety of transgene inserts, as, for example, in gene therapy.

## Materials and Methods

### Sequences, Alignments, and Evolutionary Distances

ncRNAs are commonly classified by their length into small (18–31 nt), medium (32–200 nt) and long (from 200 nt up to several hundred kilobases) ncRNAs (Wilusz et al. 2009; Nagano and Fraser 2011). The lncRNAs are the most mysterious group among those three. Few of them have been experimentally characterized and many are poorly conserved on the sequence level (Amaral et al. 2011; Lee 2012). The group of lncRNAs can be further divided into those transcripts that overlap protein-coding genes and lincRNAs (Ponting et al. 2009). The lncRNAs that overlap protein-coding genes are most likely involved in sense–antisense regulation (Chen et al. 2005). Their evolution is likely to be constrained by the evolution of the antisense target and hence is not optimal to ask about selection on ncRNAs more generally. Here then we solely examine lincRNAs. So far, few lincRNAs have been experimentally characterized, but functional lincRNAs seem to be involved in protein-coding gene regulation by means of chromatin remodeling, transcriptional control, and posttranscriptional processing (Mercer and Mattick 2013).

The data set of putative human lincRNAs identified by Cabili et al. (2011) was downloaded as BED (Browser Extensible Data, including genomic coordinates) formatted data (supplementary material in Cabili et al. 2011). These putative lincRNAs were inferred based on the reconstruction of transcripts based on greater than 4 billion RNA-seq reads collected from 24 human tissues. In total, 10,500 putative lincRNAs have been identified by the authors. This set of candidate lincRNAs was filtered to remove transcripts where evidence for protein-coding potential could be detected (as specified by the original authors), which leads to a subset of 8,195 lincRNAs. This subset was further been filtered to remove lincRNA genes that could not be reconstructed in at least two different tissues, or reconstructed by two different assemblers in the same tissue, leaving a stringent subset of 4,662 lincRNAs (Kapranov et al. 2007). Unless otherwise noted, this stringent lincRNA subset was used for analyses in this study.

The intron and exon sequences (based on the hg19 assembly) corresponding to the lincRNA BED data were downloaded from the Galaxy server (Blankenberg et al. 2011). The galaxy server was also used to extract alignments of these regions to the rhesus macaque genome (rheMac2 assembly), based on the UCSC 46-way whole-genome multiZ alignment (Kent et al. 2002). The intron and exon alignment blocks were concatenated with the “stitch gene blocks” function provided by the Galaxy server to produce alignments of concatenated exons and concatenated introns for each lincRNA gene. The fraction of alignment positions that correspond to insertions/deletions (indels) was calculated with a custom script and alignments with a fraction of indels higher than a given threshold were discarded. Unless otherwise noted, this threshold was set to 15% (this threshold, while arbitrary, enables comparison to other analyses).

To compare the properties of lincRNAs with those of protein-coding genes, we gathered BED12 data for the 17,132 reconstructed protein-coding transcripts from the data set and constructed alignments to the homologous regions in the macaque genome with the same approach as described for the lincRNAs. Note that we employ intronic sequence away from exon ends as a comparator not because all the sequence is necessarily neutrally evolving but because it 1) controls for local variation in the mutation rate (Matassi et al. 1999; Lercher et al. 2001), 2) conforms with numerous prior analyses (Hurst and Smith 1999; Pang et al. 2006), and 3) controls for transcription-coupled mutational/repair processes (Hanawalt and Spivak 2008). Importantly, comparison with flanking nontranscribed sequence, even if GC matched, does not control for this. If transcription-coupled repair is prevalent even on neutrally evolving sequence, in comparing exonic rates of evolution to flanking but untranscribed and hence unrepaired sequence, one could potentially misinfer purifying selection on the exon. In contrast, as introns may contain hidden residues under constraint, the comparison of exonic to intronic rates to infer purifying selection on the exons is most probably conservative. We note in addition that with biased gene conversion prevalent in the human genome (Duret and Galtier 2009) no sequence can be guaranteed to provide a perfect neutral proxy.

Evolutionary distances between human and macaque sequences were calculated with a custom implementation of the method proposed by Tamura and Kumar (2002). This method relaxes the assumption of substitution pattern homogeneity among lineages and thus allows for a more accurate distance estimation. Note that to enable fair comparison between protein-coding genes and lincRNAs we use the same metric for both. This is also meaningful as the dominant constraints that we are examining, splice-related selection and RNA folding, operate at the RNA rather than the protein level.

### Expression Data

We used the expression patterns of lincRNAs and protein-coding transcripts based on the supplementary tables S2 and S6 of Cabili et al. (2011). For each lincRNA, the FPKM (fragments per kilobase of exon per million fragments mapped) value for each of the 24 studied tissues was extracted. We log-normalized the FPKM values and calculated the maximum and median FPKM for each lincRNA. The expression breadth was assessed by calculating the fraction of tissues where the respective lincRNA was detectably expressed (FPKM >0).

For analysis of the expression of genes neighboring focal ncRNA genes, we used the profiles available for H1 and K562 cell lines on Encode portal, generated with Gencode V7 annotation (2012). We considered gene expression in bins flanking focal ncRNA genes. Average gene expression per bin is calculated as below:

Note here we simply consider whether a gene is expressed or not, not its absolute level.

### ESE Hexamers

We annotated putative ESE motifs in the lincRNA and protein-coding alignments by using the set of experimentally confirmed human ESE-hexamers employed in a previous study (Parmley et al. 2006) as defined by Fairbrother, Yeo, et al. (2004). These are presented in supplementary table S4, Supplementary Material online.

### RNA Folding Simulation

We used the UNAfold (Markham and Zuker 2008) software package to computationally predict the minimum energy folding of each lincRNA sequence. The “hybrid-ss-min” tool from the UNAfold package was run on each sequence with default parameters and we subsequently inferred the number of paired nucleotides from the output file. The proportion of folded nucleotides in the minimum energy RNA structure was used as a proxy for RNA-folding stability.

### Assessing CHD1-Binding Sites in Active lincRNAs

We used ENCODE Project Consortium (2012) data in the latest release to find the CHD1-binding sites for the lincRNAs that both correspond to our stringent subset of the Cabili et al.’s data and are also found in the Macaque genome. Specifically, we downloaded the broadPeak data sets for the only human cell lines for which CHD1 modifications are available—k562 and h1-hesc (available on http://genome.ucsc.edu/cgi-bin/hgFileUi?db=hg19&g=wgEncodeBroadHistone, last accessed September 1, 2014).

To calculate the density for each lincRNA, the number of CHD1’s peaks which are overlapping this lincRNA is divided by the lincRNA-length. In addition, we consider the sum breadth of CHD1 spans (as specified by ENCODE) and consider the proportion of this span to the gene length. Unless specified otherwise, analysis is on the number of CHD1 peaks per base pair. As the K562 and H1-hesc cell lines have not been considered in the data set of Cabili et al., we assessed whether lincRNAs were expressed in these cell lines based on Caltech and CSHL RNA seq data sets available from ENCODE (http://genome.ucsc.edu/cgi-bin/hgFileUi?db=hg19&g=wgEncodeCaltechRnaSeq and http://genome.ucsc.edu/cgi-bin/hgFileUi?db=hg19&g=wgEncodeCshlLongRnaSeq [last accessed September 1, 2014] respectively). Based on these data sets, we find 346 lincRNAs to be actively expressed in the H1-hesc cell line and 338 in the K562 cell line. For analysis comparing various features against intron density in lincRNAs, we employ the larger lincRNA data set of Derrien et al. (2012).

As there are multiple sequences with no CHD1 binding, we were concerned that the Mann–Whitney *U* test might be misleading. To explore this, we used a Monte Carlo simulation to test whether the enrichment of CHD1-binding sites in active sequences could be explained by chance. To do this for each cell type, we combined the active and inactive sets and randomly selected two sets: A hypothetical active set and a hypothetical inactive set, including the same number of sequences as observed in each cell line by querying ENCODE data. This was iterated 10,000 times, each time the difference between medians of CHD1 density in two sets was calculated and compared with the difference in medians observed in the real data. The number of times the median difference in hypothetical and randomly generated sets was as high or higher than the median difference was observed. In this test the unbiased estimation of the *P* of this Monte Carlo simulation is *P* = (*n* + 1)/(*m* + 1), where *n* is the number of randomization as extreme or more extreme in the difference between the two classes as seen in the real data and *m* is the number of randomizations.

### DHS-Binding Profile

DNase hypersensitive sites (DHSs) point at open chromatin segments on chromosomes. Different tissues diverge in locations of DHSs, encouraging tissue-specific gene expression patterns. The DHSs data available through ENCODE portal are generated in two production centers, University of Washington and Duke University, through a similar procedure. WashU provides two sets for K562 accessible through: http://genome.ucsc.edu/cgi-bin/hgFileUi?db=hg19&g=wgEncodeUwDnase, last accessed September 1, 2014. Duke’s data sets are accessible from: http://genome.ucsc.edu/cgi-bin/hgFileUi?db=hg19&g=wgEncodeOpenChromDnase, last accessed September 1, 2014.

## Supplementary Material

Supplementary figures S1–S9 and tables S1–S4 are available at *Molecular Biology and Evolution* online (http://www.mbe.oxfordjournals.org/).

Supplementary Data
