# Supplementary material for: Purifying Selection on Splice-Related Motifs, Not Expression Level nor RNA Folding, Explains Nearly All Constraint on Human lincRNAs
Source: Mol Biol Evol. 2014 Aug 25;31(12):3164–83. doi: 10.1093/molbev/msu249 (PMC4245815; doi:10.1093/molbev/msu249)
Supplement: Supplementary Data [file supp_31_12_3164__index.html]

Purifying selection on splice-related motifs, not expression level nor RNA folding, explains nearly all constraint on human lincRNAs. — Purifying Selection on Splice-Related Motifs, Not Expression Level nor RNA Folding, Explains Nearly All Constraint on Human lincRNAs — Purifying Selection on Splice-Related Motifs, Not Expression Level nor RNA Folding, Explains Nearly All Constraint on Human lincRNAs — Supplementary Data 

# Purifying Selection on Splice-Related Motifs, Not Expression Level nor RNA Folding, Explains Nearly All Constraint on Human lincRNAs

## Supplementary Data

files

**Files in this Data Supplement:**

- Supplementary Data - pdf file
- Supplementary Data - pdf file
- Supplementary Data - pdf file
- Supplementary Data - pdf file
- Supplementary Data - pdf file
- Supplementary Data - pdf file
- Supplementary Data - pdf file
- Supplementary Data - pdf file
- Supplementary Data - pdf file
- Supplementary Data - pdf file
- Supplementary Data - xls file
- Supplementary Data - xls file
- Supplementary Data - xlsx file
