# Supplementary material for: Purifying Selection on Splice-Related Motifs, Not Expression Level nor RNA Folding, Explains Nearly All Constraint on Human lincRNAs
Source: Mol Biol Evol. 2014 Aug 25;31(12):3164–83. doi: 10.1093/molbev/msu249 (PMC4245815; doi:10.1093/molbev/msu249)

Supplementary figure 1. Relative frequencies of bases predicted to be part of an ESE motif as a function of the distance to the nearest intron, starting at a distance of 6 (a+b). Shown in c+d is the decadic logarithm of the average intron length for lincRNA and protein-coding genes vs. the density of ESE motifs on the exon sequences of this gene. For c+d, "density" has been measured as the number of nucleotides that belong to a putative ESE motif divided by the summed length of exons for the respective gene. This figure includes all lincRNAs instead of only the conservative subset (see methods).

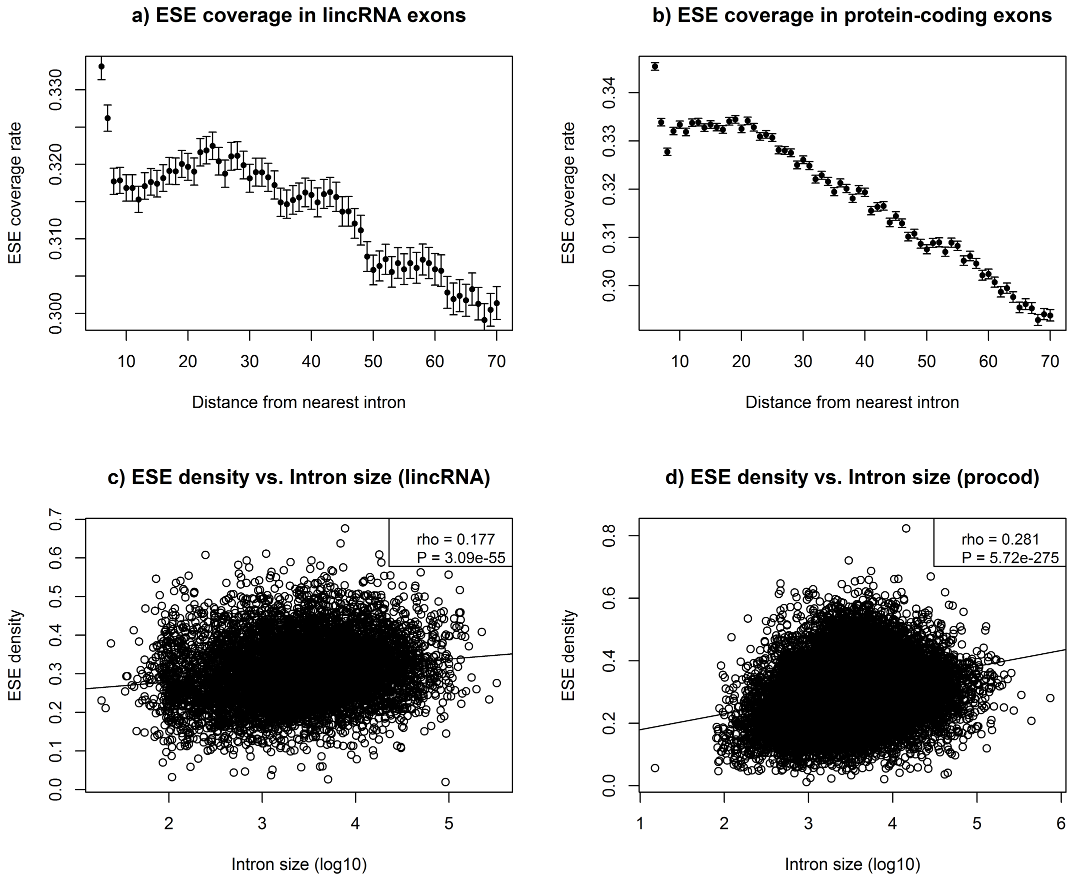

Supplement: Supplementary Data [file supp_msu249_Supplementary_figure_1.pdf]
