# Supplementary material for: Purifying Selection on Splice-Related Motifs, Not Expression Level nor RNA Folding, Explains Nearly All Constraint on Human lincRNAs
Source: Mol Biol Evol. 2014 Aug 25;31(12):3164–83. doi: 10.1093/molbev/msu249 (PMC4245815; doi:10.1093/molbev/msu249)
Supplement: Supplementary Data [file supp_msu249_Table_S1.pdf]

Table S1. Normal and partial correlations with evolutionary rate (measured as Tamura-Kumar distance, see methods) using Pearson product moment correlation and Spearmans. Numbers highlighted in bold are significant after Bonferonni correction with N=14.

lincRNA				
	Pearson		Spearman	
	Normal	Partial	Normal	Partial
Max. Expression rate	-0.025	0.003	-0.005	0.032
Med. Expression rate	-0.028	-0.011	-0.025	-0.035
Exp. breadth	-0.046#	**-0.088****	-0.038	**-0.091****
RNA stability	0.012	-0.013	0.048#	0.009
Frac70	-0.034	-0.017#	**-0.051#**	-0.011
ESE density	**-0.178*****	**-0.198*****	**-0.182*****	**-0.194*****
GC	-0.043#	**-0.096****	**-0.058***	**-0.102*****
**Protein-coding**				
	Pearson		Spearman	
	Normal	Partial	Normal	Partial
Max. expression	**-0.198*****	**0.038***	**-0.203*****	-0.019
Med. Expression	**-0.302*****	**-0.087*****	**-0.339*****	**-0.063*****
Exp. Breadth	**-0.381*****	**-0.255*****	**-0.369*****	**-0.189*****
RNA stability	**0.038***	**-0.04****	**0.154*****	**0.028***
Frac70	**-0.201*****	**-0.092*****	**-0.222*****	**-0.101*****
ESE density	**-0.22*****	-0.023#	**-0.29*****	0.008
GC	**0.219*****	**0.113*****	**0.313*****	**0.168*****

Significance codes for p-values: # $P < 0.01$; * $P < 10^{-3}$; ** $P < 10^{-6}$; *** $P < 10^{-9}$
