# Supplementary material for: Purifying Selection on Splice-Related Motifs, Not Expression Level nor RNA Folding, Explains Nearly All Constraint on Human lincRNAs
Source: Mol Biol Evol. 2014 Aug 25;31(12):3164–83. doi: 10.1093/molbev/msu249 (PMC4245815; doi:10.1093/molbev/msu249)

Supplementary Figure 2. ESE motifs evolve slower than non-ESE sites. The substitution rates in ESEs and non-ESEs are shown as a function of the distance from the nearest splice-junction. This figure includes all lincRNAs instead of only the conservative subset (see methods).

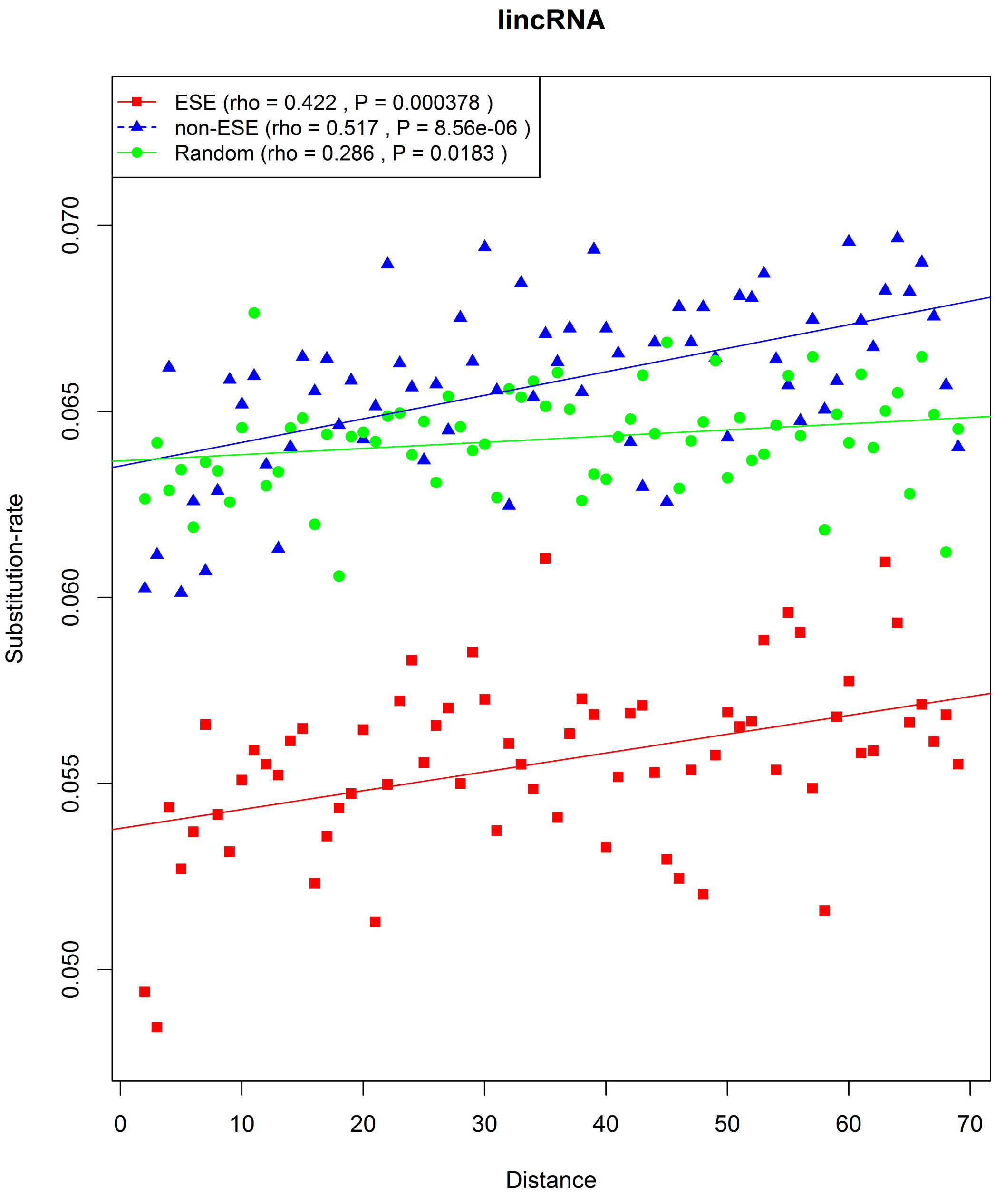

Supplement: Supplementary Data [file supp_msu249_Supplementary_Figure_2.pdf]
