# Supplementary material for: Purifying Selection on Splice-Related Motifs, Not Expression Level nor RNA Folding, Explains Nearly All Constraint on Human lincRNAs
Source: Mol Biol Evol. 2014 Aug 25;31(12):3164–83. doi: 10.1093/molbev/msu249 (PMC4245815; doi:10.1093/molbev/msu249)

Supplementary Figure 3. Exon cores and flanks evolve at different rates. The distributions of *Kec/Kic* and *Kef/Kec* values are shown for protein-coding genes and lincRNAs. This figure includes all lincRNAs instead of only the conservative subset (see methods).

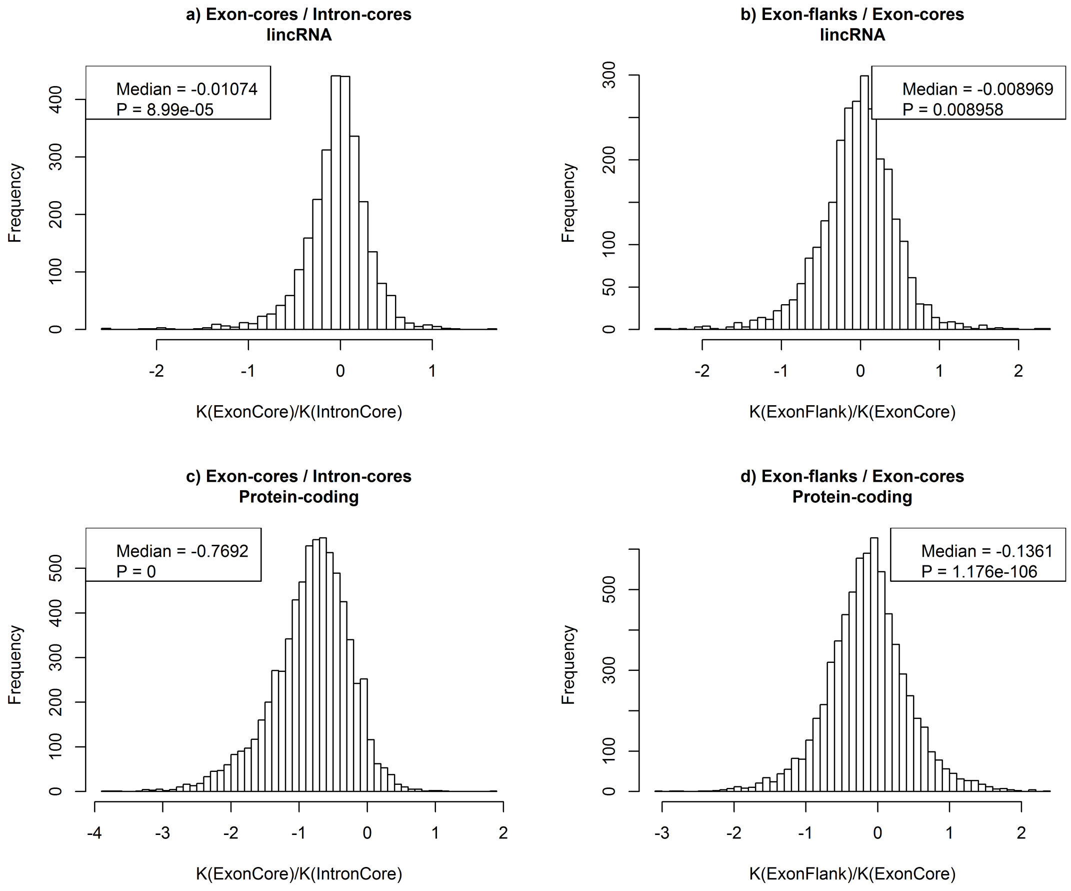

Supplement: Supplementary Data [file supp_msu249_Supplementary_Figure_3.pdf]
