# Supplementary material for: Purifying Selection on Splice-Related Motifs, Not Expression Level nor RNA Folding, Explains Nearly All Constraint on Human lincRNAs
Source: Mol Biol Evol. 2014 Aug 25;31(12):3164–83. doi: 10.1093/molbev/msu249 (PMC4245815; doi:10.1093/molbev/msu249)

Supplementary Figure 4. Evolutionary rates and lincRNA expression. The evolutionary distance to the macaque homologue was plotted vs the values of maximum expression (a), median expression (b) and expression breadth (c) for each lincRNA. This figure includes all lincRNAs instead of only the conservative subset (see methods).

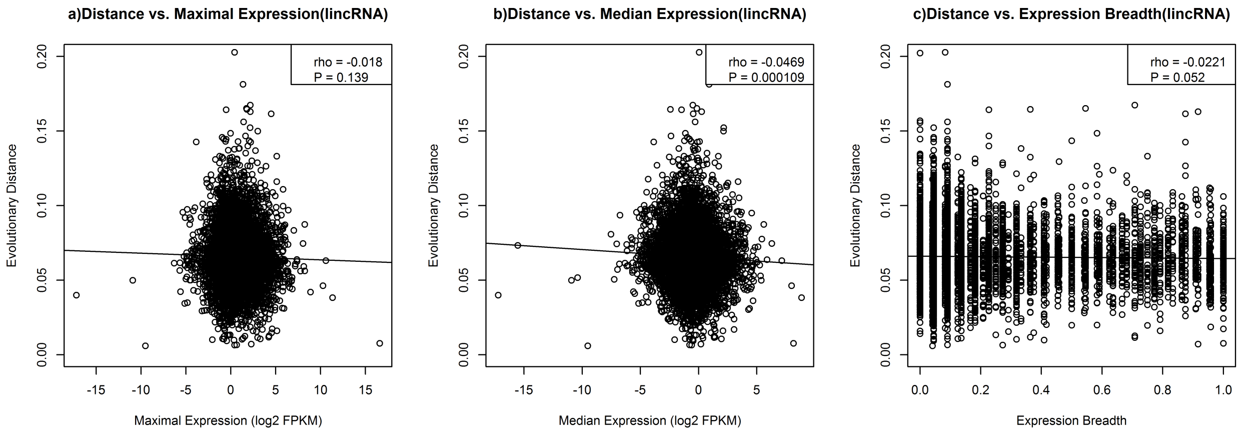

Supplement: Supplementary Data [file supp_msu249_Supplementary_Figure_4.pdf]
