# Supplementary material for: Purifying Selection on Splice-Related Motifs, Not Expression Level nor RNA Folding, Explains Nearly All Constraint on Human lincRNAs
Source: Mol Biol Evol. 2014 Aug 25;31(12):3164–83. doi: 10.1093/molbev/msu249 (PMC4245815; doi:10.1093/molbev/msu249)

Supplementary Figure 5

Might the lower rate of evolution of ESE be an artifact of dataset contamination with protein coding exons? To address this we divided the internal exons into those with stop codons in all three frames (unlikely to be protein coding) and all the others. 2667 out of 4662 conservative lincRNAs are encoded by three or more exons, out of those 2667, 2080 have stop codons in all three frames. These we term the allstop exons. The others we call the nonstop. As can be seen from the figures below, these two classes of exon are effectively identical in absolute rates in and our of ESE. There is a 15% difference in the rate of evolution of ESE to nonESE in both divides of the data as there is at 4 fold degenerates sites in ESEs in protein coding exons.

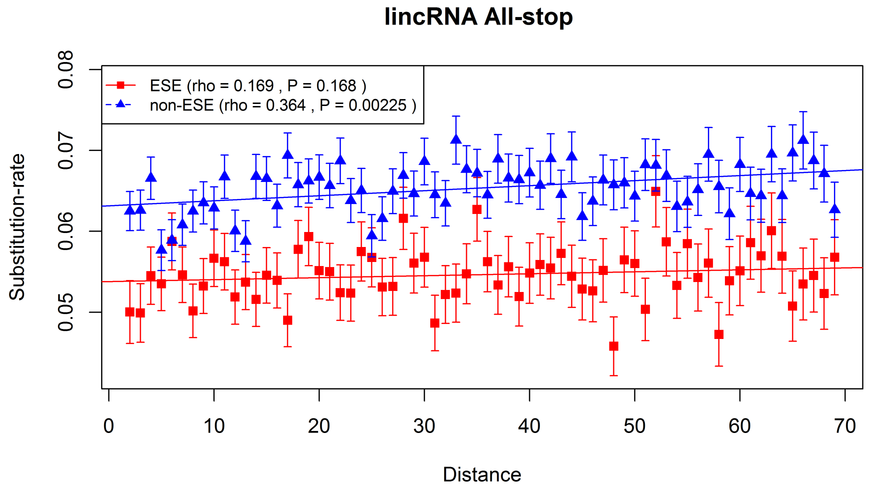

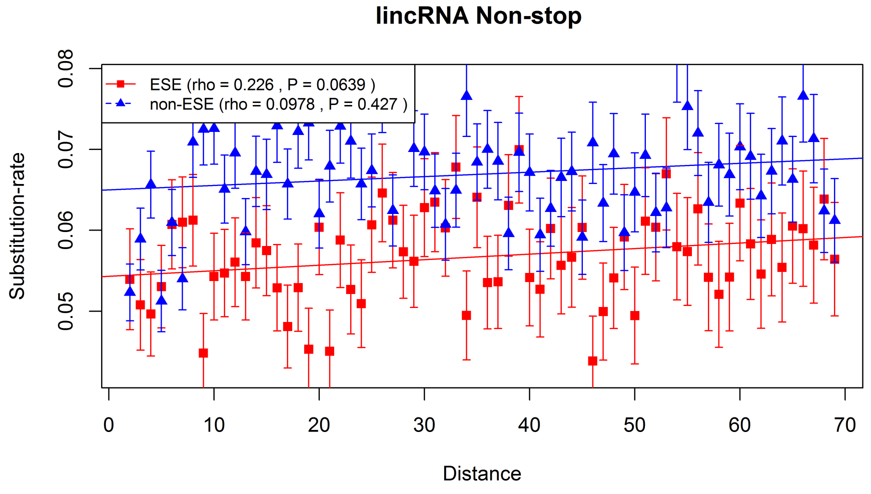

Supplement: Supplementary Data [file supp_msu249_Supplementary_Figure_5.pdf]
