# Supplementary figures and images for: Purifying Selection on Splice-Related Motifs, Not Expression Level nor RNA Folding, Explains Nearly All Constraint on Human lincRNAs
Source: Mol Biol Evol. 2014 Aug 25;31(12):3164–83. doi: 10.1093/molbev/msu249 (PMC4245815; doi:10.1093/molbev/msu249)

H1 – WashU

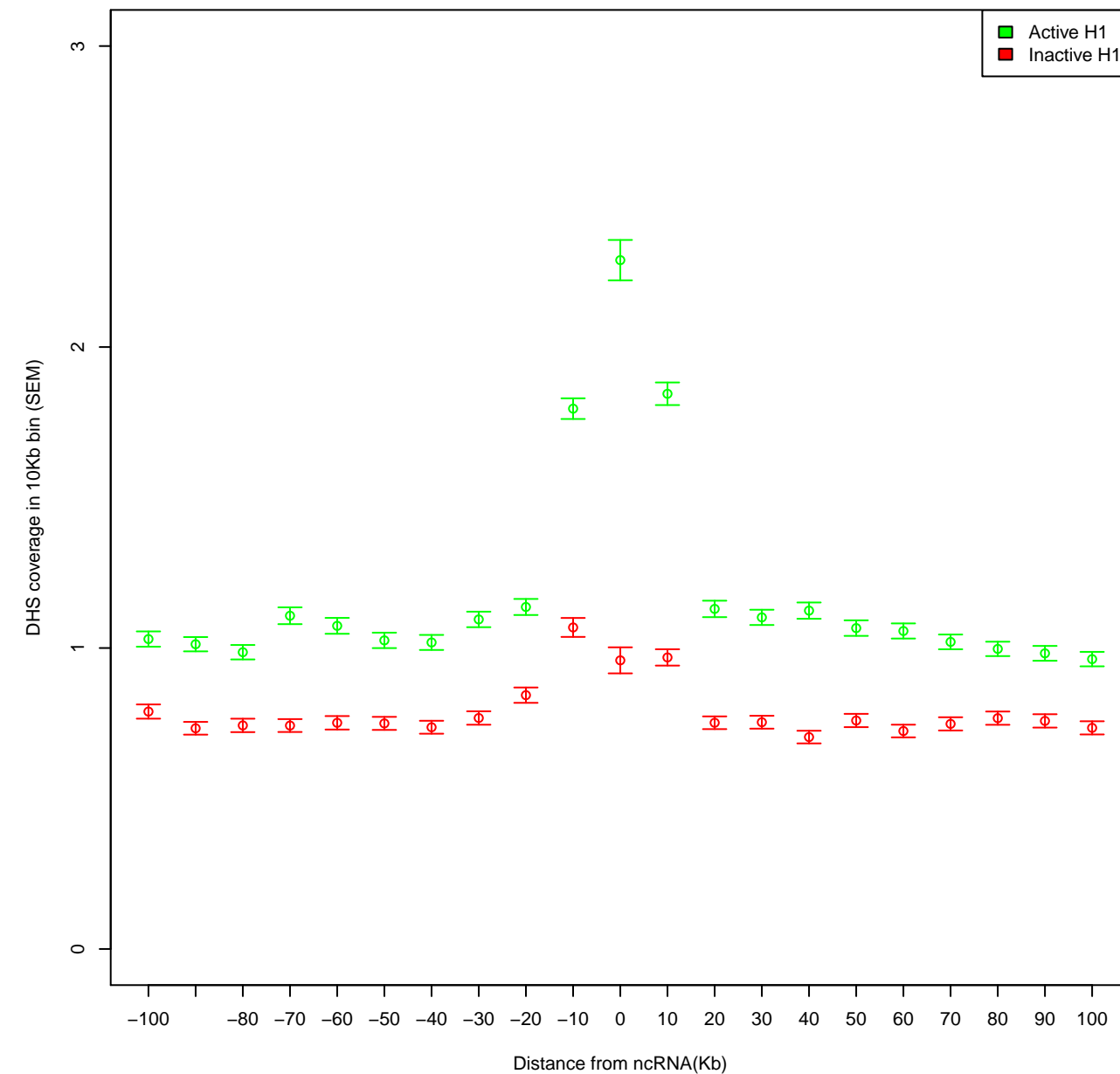

K562 Rep1 – WashU

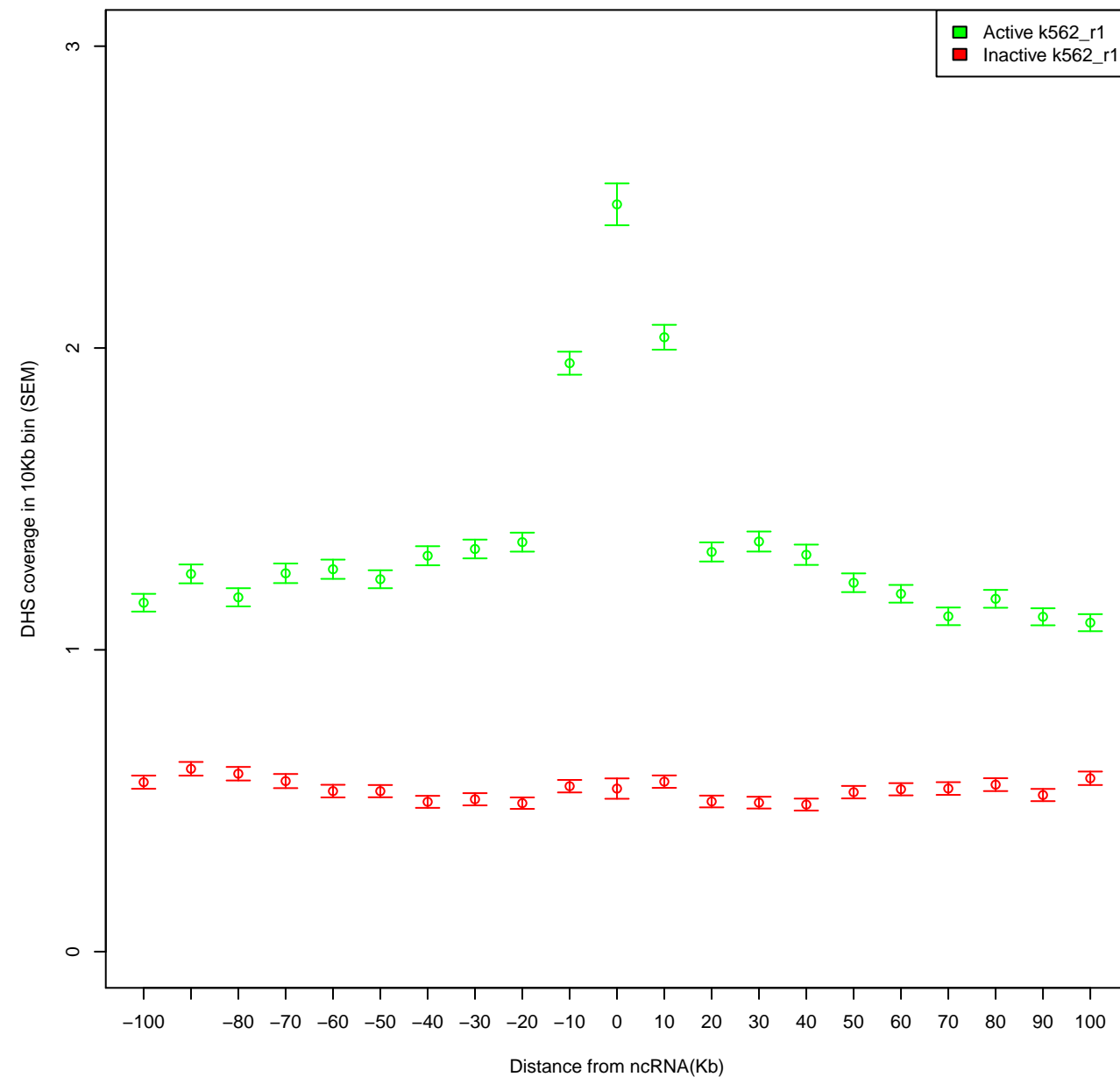

K562 Rep2 – WashU

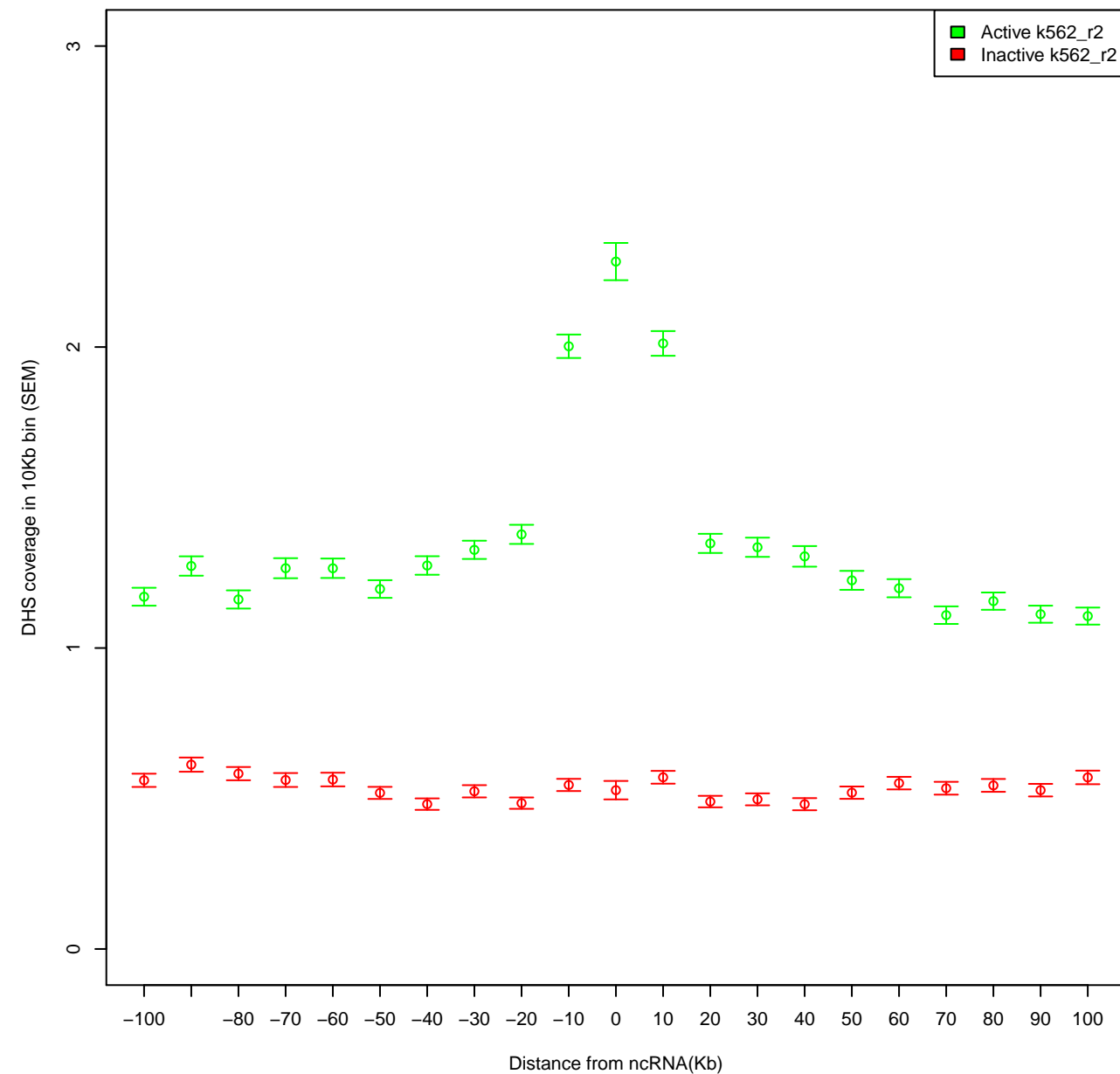

Supplement: Supplementary Data [file supp_msu249_Supplementary_Figure_6.pdf]

H1 – WashU

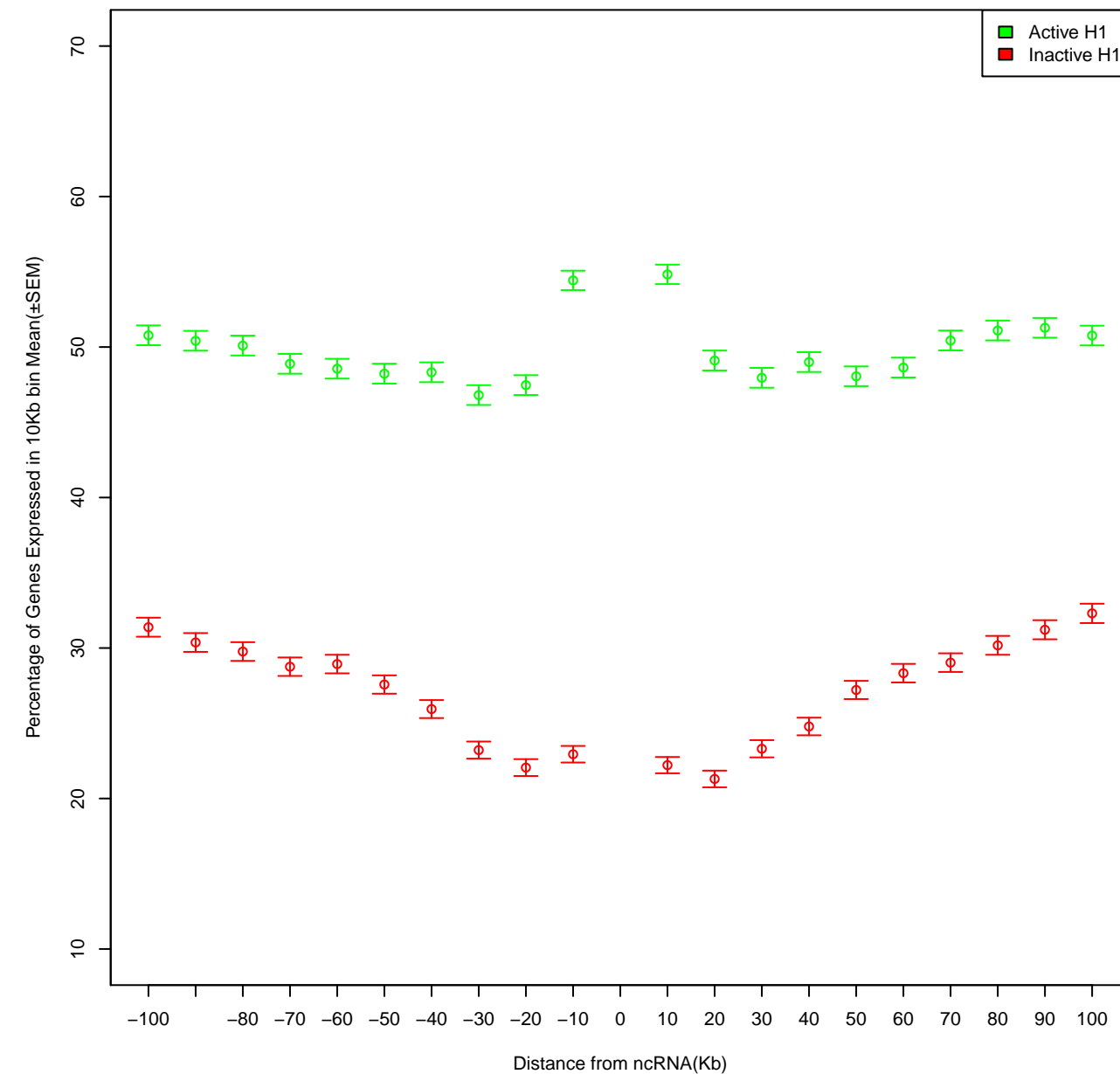

K562 Rep1 – WashU

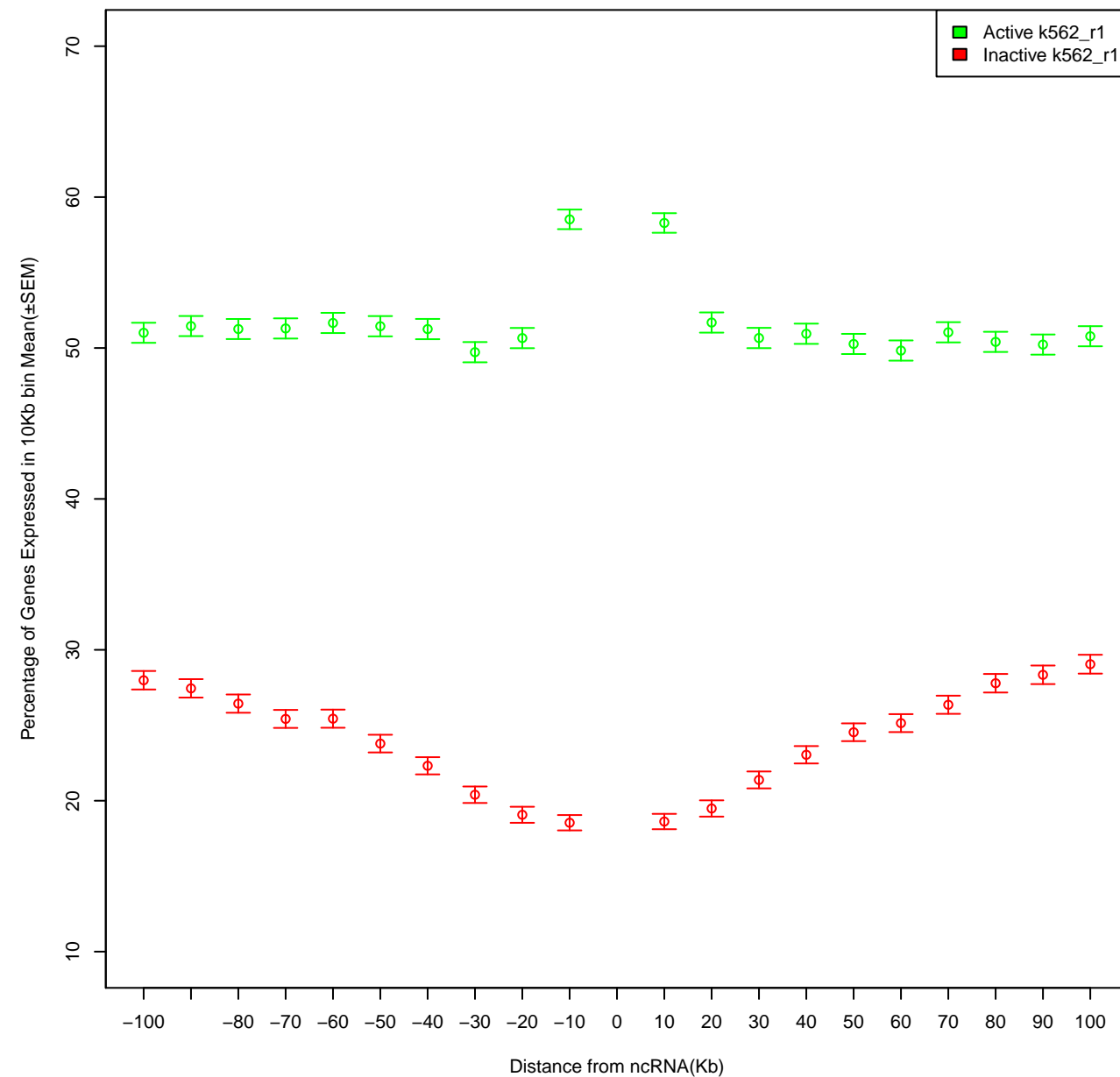

K562 Rep2 – WashU

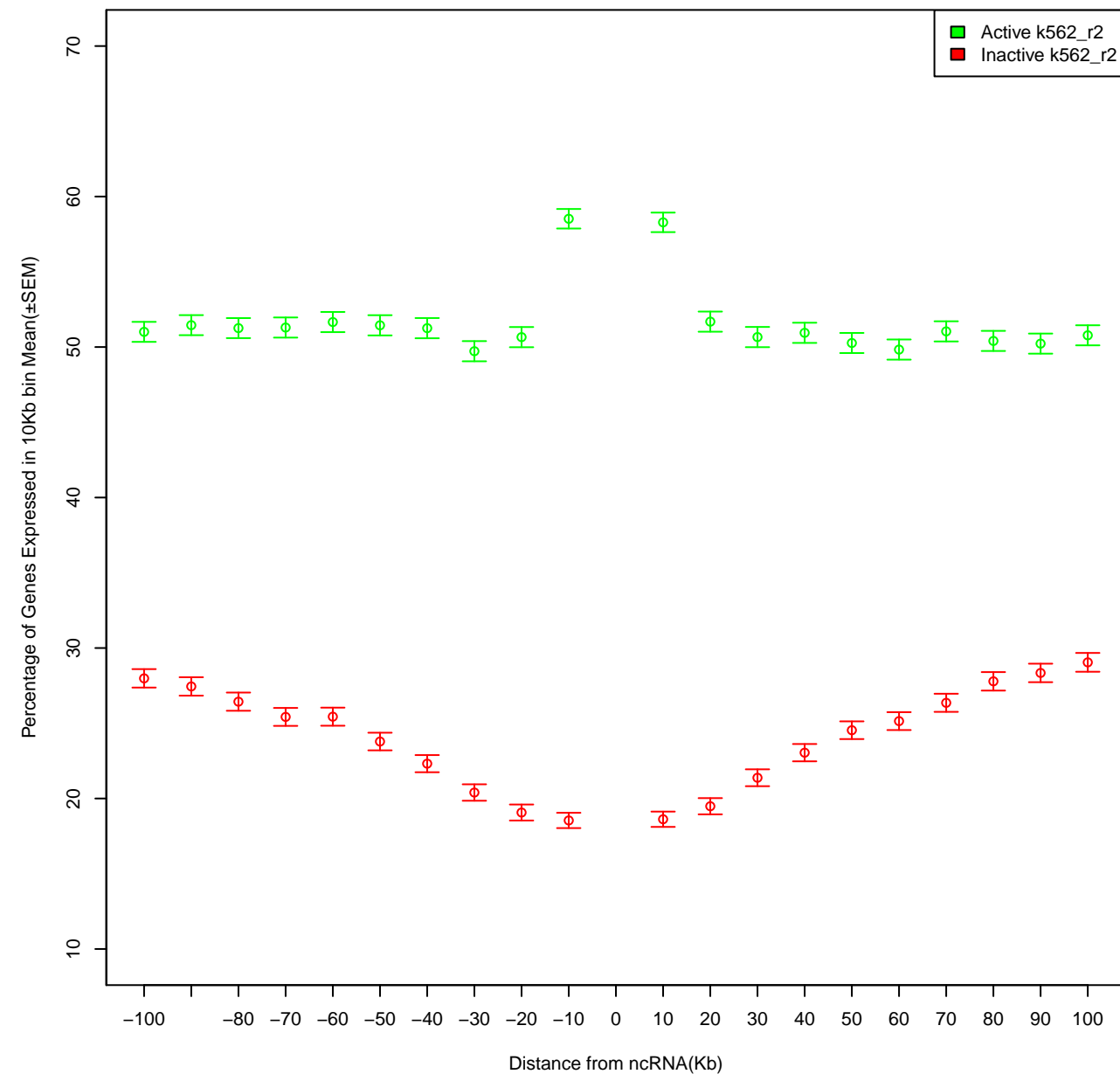

Supplement: Supplementary Data [file supp_msu249_Supplementary_Figure_8.pdf]
