# Supplementary material for: Purifying Selection on Splice-Related Motifs, Not Expression Level nor RNA Folding, Explains Nearly All Constraint on Human lincRNAs
Source: Mol Biol Evol. 2014 Aug 25;31(12):3164–83. doi: 10.1093/molbev/msu249 (PMC4245815; doi:10.1093/molbev/msu249)

Supplementary Figure 7. The relationship between local DHS density (+/-50kb either side of a focal gene) and the intron density of that gene.

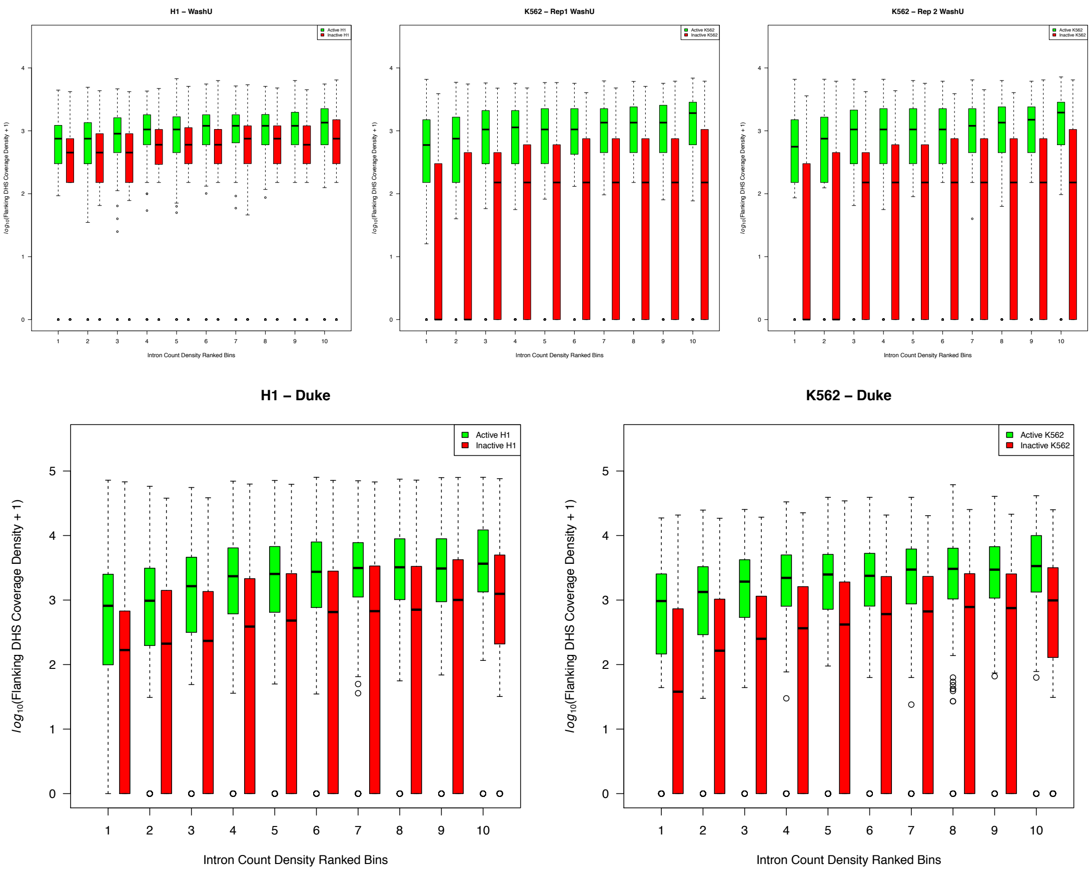

Supplement: Supplementary Data [file supp_msu249_Supplementary_Figure_7.pdf]
