# Supplementary material for: Purifying Selection on Splice-Related Motifs, Not Expression Level nor RNA Folding, Explains Nearly All Constraint on Human lincRNAs
Source: Mol Biol Evol. 2014 Aug 25;31(12):3164–83. doi: 10.1093/molbev/msu249 (PMC4245815; doi:10.1093/molbev/msu249)

Supplementary Figure 9. The relationship between the local expression (proportion of genes expressed in +/- 50kb window) and the intron density of the focal gene.

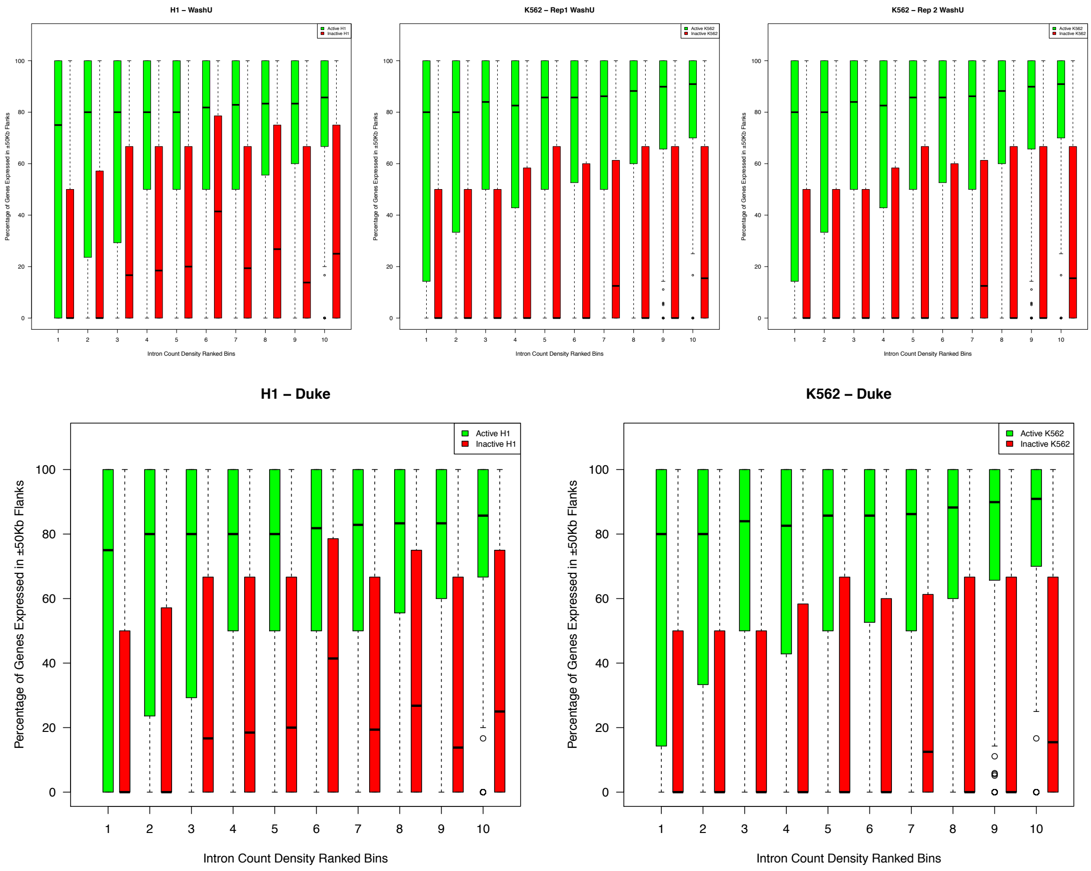

Supplement: Supplementary Data [file supp_msu249_Supplementary_Figure_9.pdf]
